# Nlp-dependent ER-to-Golgi transport

**DOI:** 10.7150/ijbs.91792

**Published:** 2024-05-11

**Authors:** Danna Yeerken, Wenchang Xiao, Jia Li, Yan Wang, Qingnan Wu, Jie Chen, Wei Gong, Mengzhu Lv, Ting Wang, Ying Gong, Rui Liu, Jiawen Fan, Jinting Li, Weimin Zhang, Qimin Zhan

**Affiliations:** 1 Key laboratory of Carcinogenesis and Translational Research (Ministry of Education/Beijing), Laboratory of Molecular Oncology, Peking University Cancer Hospital & Institute, Beijing 100142, China.; 2 Department of Biomedical Engineering, College of Life Science and Technology, Huazhong University of Science and Technology (HUST), Wuhan, China.; 3 Research Unit of Molecular Cancer Research, Chinese Academy of Medical Sciences, Beijing, China.; 4 Institute of Cancer Research, Shenzhen Bay Laboratory, Shenzhen 518107, China. Department of Oncology, Cancer Institute, Peking University Shenzhen Hospital, Shenzhen Peking University-Hong Kong University of Science and Technology (PKU-HKUST) Medical Center, Shenzhen 518035, China.; 5 Peking University International Cancer Institute, Beijing 100191, China.; 6 Soochow University Cancer Institute, Suzhou 215127, China.

**Keywords:** Nlp, Adapter, ER-to-Golgi cargo trafficking, ER stress, B cell lymphoma

## Abstract

The mechanism that maintains ER-to-Golgi vesicles formation and transport is complicated. As one of the adapters, Ninein-like protein (Nlp) participated in assembly and transporting of partial ER-to-Golgi vesicles that contained specific proteins, such as β-Catenin and STING. Nlp acted as a platform to sustain the specificity and continuity of cargoes during COPII and COPI-coated vesicle transition and transportation through binding directly with SEC31A as well as Rab1B. Thus, we proposed an integrated transport model that particular adapter participated in specific cargo selection or transportation through cooperating with different membrane associated proteins to ensure the continuity of cargo trafficking. Deficiency of Nlp led to vesicle budding failure and accumulation of unprocessed proteins in ER, which further caused ER stress as well as Golgi fragmentation, and PERK-eIF2α pathway of UPR was activated to reduce the synthesis of universal proteins. In contrast, upregulation of Nlp resulted in Golgi fragmentation, which enhanced the cargo transport efficiency between ER and Golgi. Moreover, *Nlp* deficient mice were prone to spontaneous B cell lymphoma, since the developments and functions of lymphocytes significantly depended on secretory proteins through ER-to-Golgi vesicle trafficking, including IL-13, IL-17 and IL-21. Thus, perturbations of Nlp altered ER-to-Golgi communication and cellular homeostasis, and might contribute to the pathogenesis of B cell lymphoma.

## Introduction

The transfer of substances between various parts of the cell's inner membrane system is usually carried out by vesicle transportation, a highly organized, directionally transported, and specifically targeted system 1, 2. As the basic trafficking networks in cells, there are two representative cage-like coat protein complexes that mediate endoplasmic reticulum (ER) to Golgi transport pathway: COPII and COPI-coated vesicles 3. COPII and COPI-coated vesicles transport multiple proteins participating in cell signaling 4, 5, membrane remodeling 6, 7, protein-protein interaction 8 and secretory pathway 9, 10, such as cytokines, insulins, growth factors, hormones and extracellular matrix proteins. The disorder of ER-to-Golgi vesicle transport causes various diseases, including immune system disfunction 11, nervous system disease 12, diabetes 5, 13 and cancer 14. ER-to-Golgi transportation begins with the formation of COPII-coated vesicle in ER-exit site (ERES) 15. The recruitment of small GTP binding protein (GTPase) SAR1 initiates the assembly of COPII-coated vesicles, and then heterodimeric complexes SEC23/SEC24 and SEC13/SEC31 are recruited in turn to form their inner and outer protein coats 16. However, the integration and transportation of ER-to-Golgi vesicles after forming of COPII-coated vesicles remain unclear.

Different models are proposed to attempt to explain the trafficking pathways of COPII and COPI-coated vesicles 17. Unfortunately, research data about these finely organized processes are conflicted. Some studies have shown that COPII-coated vesicles were responsible for ER-to-Golgi cargoes trafficking independently, while other studies supported COPII and COPI-coated vesicles participated in ER-to-Golgi cargoes transportation in succession 16-19. In addition, the existence of microtubules during COPII-coated vesicle transportation was also controversial. Different data verified COPII-coated vesicles diffused to ERGIC or transported by microtubules to ERGIC as well as Golgi 20. Another ambiguity is whether ERGIC is indispensable during ER-to-Golgi vesicle transport 15, 21. Recently, Weigel et al. demonstrated that ERES formed a receptacle which was still linked to the ER by a thin neck. COPII-coated vesicles localized to the neck of ERES and regulated the entrance of cargoes to ERES, while COPI-coated vesicles transported ER-to-Golgi cargoes in succession through microtubules 15. Though this model explains ER-to-Golgi vesicle trafficking to a great extent, the question of how to maintain the specificity, continuity and efficiency of cargoes during multi-step transport processes remains unknown.

Increasing evidences have highlighted there were ER-to-Golgi vesicle transport related proteins controlling the specificity of ER-to-Golgi cargoes 22. So far, several proteins have been found to modulate formation or transport of vesicles. As the essential components of COPII-coated vesicles, SEC24 bound to cargoes, including E-cadherin, various compound transporters, neurotransmitters, noradrenaline and others, through different sequence motifs or conformational motifs in ERES 23-26. Transport and Golgi organization 1 (Tango1) formed macromolecular complexes in ERES to collect collagens through regulating the size of COPII-coated vesicles 27. Moreover, Syntaxin 5 (STX5) participated in pro-collagens and low-density lipoproteins sorting in ERES 28, 29. Unfortunately, various aspects of this cargo-sorting pattern remain unclear. Most of these studies focused on one or some proteins collected in ERES by COPII-coated vesicles, while overlooking the cargo integrity of the COPII and COPI-coated vesicles during the continuous trafficking processes or the classification of the specific proteins. Thus, the ER-to-Golgi trafficking network is an essential issue to explore. The proposition of activating adapters 2, 30 inspired us to explore the ER-to-Golgi vesicle transport specificity as a continuous process. Recent studies have shown that dynein interacted with different activating adapters, which acted as switches of trafficking or dynein-dynactin complexes stability maintainer, to transport cargoes through microtubules 31. Interestingly, Ninein-like protein (Nlp) was found to participate in materials transport as an adapter 32, 33. Nlp is a well-known microtubule-related protein that participates in most steps of mitosis 34, 35 or functions as binding platforms of Rab7 or Rab8-vesicles in autophagosome or photoreceptor respectively 36, 37. Therefore, Nlp might be an essential regulator in intracellular vesicle trafficking system, which still needs to be further established.

In this study, we utilized SEC31A-TurboID system to explore the potential adapters for ER-to-Golgi vesicles, and found that Nlp could act as a platform during the formation of COPII-coated vesicles, and cooperate with membrane protein Rab1B as well as dynein-dynactin complex to maintain the continuity of cargoes during COPII-COPI coat transition and transportation. We also analyzed cargo specificity of Nlp binding vesicles using split-TurboID and TurboID tools, and found that Nlp selected or transported specific cargoes, such as STING and β-Catenin. The alterations of Nlp resulted in morphology and communication manner changes of ER and Golgi. Subsequently, we demonstrated that perturbation of Nlp was related to abnormal secretion of cytokines and B cell development, which could contribute to the spontaneous B cell lymphoma in *Nlp* deficient mice.

## Materials and Methods

### Cell culture and transfection

Mouse embryonic fibroblast of experimental groups MEF-*Nlp*^+/-^, MEF-*Nlp*^-/-^ and the wild type control groups MEF-*Nlp*^+/+^, HEK293T, HeLa and HeLa derived cell lines (stable control HeLa-C3 and HeLa-EGFP-Nlp cells; Golgi-mCherry HeLa cells (Golgi-mCherry expressed the N-terminal 82 amino acids of human B4GALT1 gene)) were cultured in Dulbecco's modified Eagle's medium (DMEM) supplemented with 10% fetal bovine serum (FBS) and 1% penicillin/streptomycin, at 37°C with 5% CO_2_. KYSE450 were cultured with PRIM-1640 medium supplemented with 10% FBS and 1% penicillin/streptomycin, at 37°C with 5% CO_2_. Transient transfection of siRNA knockdown was performed using Lipofectamine 2000 reagent (Invitrogen) according to manufacturer's instruction. Transient transfection of plasmid overexpression was carried out by Neofect DNA transfection reagent (NEOFECT) or Lipofectamine 2000 reagent (Invitrogen) directed by manufacturer's instruction. Cultured medium of all transient transfected cells was changed to fresh medium after 6 hours of transfection. Cells were harvested 36-60 hours post transfection.

### Immunoblotting

Cells were collected and lysed in cold lysis buffer (50 mM Tris, pH 7.4, 150 mM NaCl, 1% Triton X-100) in the presence of protease inhibitor and phosphatase inhibitor (Roche) for 30 min on ice. Quantified total protein concentration by BCA protein assay kit (ThermoFisher Scientific, USA). Proteins were separated by 6%-12% SDS-PAGE and transferred to polyvinylidene difluoride membrane (0.22 μm or 0.45 μm). After 5% bovine serum albumin (BSA) incubation, membrane was incubated with primary antibodies and secondary antibodies conjugated with horseradish peroxidase (HRP). Chemiluminescence signals were detected with Amersham Imager 600.

### Co-IP

Cells were collected and lysed with cold 1% NP-40 buffer (20 mM Tris, pH=7.4, 100 mM NaCl, 20 mM KCl, 1.5 mM MgCl_2_, 1% NP-40, protease inhibitors cocktail (Roche)). Pre-incubated Protein A/G Magnetic Beads (MCE) with ~2 μg IgG or antibody at 4°C for an hour, then incubated with cell lysis at 4°C overnight. The precipitates were washed with 1% NP-40 buffer for five times before preparation for immunoblotting assay.

### GST pull-down

GST-tag fusion proteins as well as GST protein were purified by Glutathione High Capacity Magnetic Agarose Beads (Sigma) at 4°C overnight. Glutathione Magnetic Agarose Beads-conjugated GST fusion proteins were mixed with 1 mg Nlp-6*His fusion protein for 6 hours at 4°C. Beads were washed seven times with 1% NP-40 buffer before boiled in loading buffer. The purified proteins were verified by Coomassie brilliant blue, silver staining and immunoblotting. The binding proteins were analyzed by immunoblotting assay.

The GST-tag of SEC31A-GST and Rab1B purified proteins were removed by Thrombin (0.3 U/100 μg) overnight at 4°C or 2 hours at room temperature, and spun down by ultrafiltration spin column. The 70 kD protein expressed by *E. coli* dnaK gene was removed by ATP-containing buffer (50 mM Tris-HCl, 2 mM ATP, 10 mM MgSO4, pH 7.4) at 37ºC for 10 min.

### SPR

The binding affinities of Nlp-6*His for its target purified proteins SEC31A and Rab1B were assayed by Biacore 8 K instrument (GE Healthcare) for surface plasmon resonance (SPR). The CM5 sensor chip was used to immobilize 6000 RU of Nlp-6*His purified protein to the sensor surface by the standard amine coupling reaction in PBS running buffer at 25 °C. Gradient concentrations of SEC31A or Rab1B purified protein were injected into the channels to evaluate the binding affinity. Biacore 8 K Evaluation Software was used to calculate the dissociation constants (KD values).

### Confocal microscopy

For different cell lines, cells were grown on cover glass slides in confocal dishes (Nest Scientific) for 12-48 hours. After washing with PBS, cells were fixed with 4% paraformaldehyde for 10 min. Cells were washed with PBS and permeabilized with 0.2% TritonX-100 for 10 min and blocked with goat serum for an hour. Cells were then incubated overnight with appropriate primary antibodies at 4°C. After washing with PBS, cells were incubated with Alexa-Fluor-conjugated secondary antibodies for an hour and DAPI for 10 min. For *Nlp*^+/+^, *Nlp*^+/-^ and *Nlp*^-/-^ mice, mice livers and spleens were made to 5 μm thick frozen slices by CM1950 (Leica). Tissues were fixed with 4% paraformaldehyde for 20 min, penetrated with 0.5% TritonX-100 for 5 min and blocked with goat serum for an hour. Tissues were incubated overnight with appropriate primary antibodies at 4°C and incubated with Alexa-Fluor-conjugated secondary antibodies for an hour. Images were taken with Zeiss LSM780, Zeiss LSM880, Zeiss LSM980, Leica SP8 STED, Leica SP8 DIVE or Leica Mica. For time lapse, live cells were imaged in confocal dishes in live cell workstation of LSM780 at 37°C with 5% CO_2_.

### PLA

Cells were cultured in confocal dishes. After 48 hours of siRNA knockdown or plasmid transiently transfected by Lipofectamine 2000 reagent (Invitrogen), Duolink PLA Probes, Duolink Fluorescent Detection Reagent, Wash Buffers for Fluorescence and Duolink Mounting Media with DAPI (Sigma-Aldrich) were used according to manufacturer's instruction for the immunofluorescent detection.

### RUSH

RUSH assay was performed as previously reported 15. HeLa cell lines were transiently transfected with Str-KDEL_ST-SBP-EGFP by Lipofectamine 2000 reagent (Invitrogen). About 36 hours after transfection, cells were incubated with 80 μM of biotin for different time (0-60 min) at 37°C before fixed for Immunofluorescence staining.

### FRAP

HeLa cells were cultured and co-transfected transiently with Nlp-Myc or NC-Myc, in combination of EGFP-TFG plasmids in confocal dishes. NC-Myc was used as a negative control to overexpression of Nlp-Myc, and Myc-tag encoded “EQKLISEEDL” sequences. After 36 hours of transfection, time-lapse and photobleaching were performed by LSM780. EGFP-TFG foci was imaged and subject to photobleaching using kex=488 nm at 100% laser power until intensity drop to 20%, and images were collected every 1s to draw fluorescence recovery curve.

### STORM

HeLa cells were cultured in confocal dishes for 12 hours. Cells were fixed with 4% paraformaldehyde for 10 min, permeabilized with 0.4% TritonX-100 for 5 min and blocked with goat serum for an hour. Incubated cells overnight with Nlp (Rabbit, 1:100) and SEC31A (Mouse, 1:50) or SEC24A (Mouse, 1:50) primary antibodies respectively at 4°C. Then cells were incubated with Alexa-Fluor-488 conjugated mouse secondary antibody and Alexa-Fluor-647 conjugated rabbit secondary antibody for an hour. Cells were imaged by A1 N-SIM STORM (NIKON), and analyzed by NIS.

### LC-MS/MS

For purified Golgi analysis, stable control HeLa-C3 and HeLa-EGFP-Nlp cells were collected and counted precisely for three times independently. Then HeLa-C3 and HeLa-EGFP-Nlp cells were divided into three equal parts, and we took out the same volume of each part to analyze total protein concentration by BCA assay three times independently. Native Golgi apparatus of Golgi were enriched with Minute™ Golgi Apparatus Enrichment Kit (Invent) in each three equal parts of stable control HeLa-C3 and HeLa-EGFP-Nlp cells. Then Golgi apparatus were lysed with cold 1% NP-40 lysis buffer and ultrasonic wave respectively and detected protein concentration by BCA assay for three times independently to select two samples nearly equal before measured by Orbitrap Fusion Lumos Tribrid LC-MS (Thermo).

For the SEC31A-TurboID system, cells were incubated without biotin (control) or with 50 μM biotin for 10 min, then cells were washed for 5 times and collected. For the split-TurboID system, cells were incubated without biotin & rapamycin (control), with 50 μM biotin, or 50 μM biotin & 100 nM rapamycin for 4 hours, then cells were washed for 5 times and collected. After fully lysing cells, cell lysis was incubated with Thermo Scientific Pierce Streptavidin Magnetic Beads (Thermo) overnight. Beads were washed and treated as described, and then analyzed by label-free mass spectrometry. Three independent replicates were performed for each group.

### *In vitro* vesicle formation assay

Cells were permeabilized with 40 mg/mL digitonin in cold KOAc buffer (110 mM potassium acetate, 2.5 mM magnesium acetate, 20 mM Hepes, pH 7.2) for 5 min. The permeabilized cells were collected by centrifugation at 4°C. After washing permeabilized cells in KOAc buffer twice, cells were resuspended in 1.5 mg/mL HeLa cell lysis (in KOAc buffer, using liquid nitrogen to lyse cells), and incubated at 30°C with 200 µM GTP and an ATP regeneration system (1 mM ATP, 40 mM creatine phosphate, 0.2 mg/mL of creatine phosphokinase) for 1 hour. Then reaction mixture was centrifuged at 14000 × g at 4°C for 20 min. The supernatant fraction containing the released vesicles was resuspended in 35% Opti-Prep and overlaid with 30% Opti-Prep. 100 µL KOAc buffer was added on the top of the step gradient of Opti-Prep. The Opti-Prep gradients were then centrifuged at 100000 g in Optima L-XP (Beckman) using SW55Ti rotor at 4°C for 90 min. After centrifugation, the top fraction was collected and analyzed by negative stain electron microscope, and immunoblotting.

### Microtubule co-sedimentation assay

As reported previously 38, cells were lysed on ice in PIPES buffer (80 mM PIPES, pH 6.8, 1 mM MgCl2, 1 mM EGTA, 100 mM NaCl, 1% Triton X-100 and protease inhibitors) for 30 min. Then cell lysis was centrifugated at 20,000g for 20 min at 4°C twice. The supernatant was supplemented with 1 mM GTP, ATP regeneration system (1 mM ATP, 40 mM creatine phosphate, 0.2 mg/mL of creatine phosphokinase) and 40 μM Taxol and incubated at 4°C or 37°C for 30 min for tubulin polymerization. Then the mixtures were centrifugation at 20,000 × g for 30 min at 4°C or 37°C respectively. The resulting pellets (P) and supernatants (S) were collected and analyzed by immunoblotting.

### Mice embryonic fibroblast genotyping

For mice, the tail tips of new born mice were cut for genotyping in the 10th day after birth. Then tissue genotypes were retested before immunoblotting or confocal. For mouse embryonic fibroblast, cells were used to analyze genotypes. TIANamp Genomic DNA Kit (TIANGEN) was used for DNA extraction.

### H&E staining and Immunohistochemistry

Sections were deparaffinized and rehydrated by graded ethanol. After treatment with EDTA antigenic retrieval buffer to retrieve antigen, sections were treated with 3% hydrogen peroxide to quench endogenous peroxidase (HRP) activity. Then nonspecific bindings were blocked by 1% goat serum albumin. Sections were incubated with appropriately diluted primary antibody overnight at 4°C. After washing in PBS, sections were treated with goat anti-mouse/rabbit IgG HRP-polymer. Then 3,3′-Diaminobenzidine was used for color developing. Slides were scanned using Zeiss Mirax Slide Scanner, and photomicrographs were examined using Aperio ImageScope (Leica Biosystems).

### Single-cell analysis

For 10× Genomics scRNA-seq, we used bone marrow, spleen, lymph node and peripheral blood of wildtype mice and *Nlp^-/-^* mice. RNA-seq data were corrected by DoubletFinder and Harmony. Data was analyzed by scMCA and Seurat.

### Inhibitors

Tunicamycin: 0.5 μg/mL for 12 hours or 2.5 μg/mL 2 hours; GSK2606414: 6 μM for 12 hours; Cycloheximide: 1 μg/mL; Nocodazole: 5 μg/mL for 2 hours; Trimethoprim: 10 μM; Tetraphenylethene maleimide (TPE-MI): 50 μM for 30 min.

### Statistics

R software and GraphPad Prism 8 were used for statistical analysis. All data were acquired from at least three independent experiments. The reported statistical significance levels were all two-sided, and p<0.05 was considered as statistically significant.

## Results

### Nlp interacted with ER-to-Golgi vesicles

**Figure 1:**
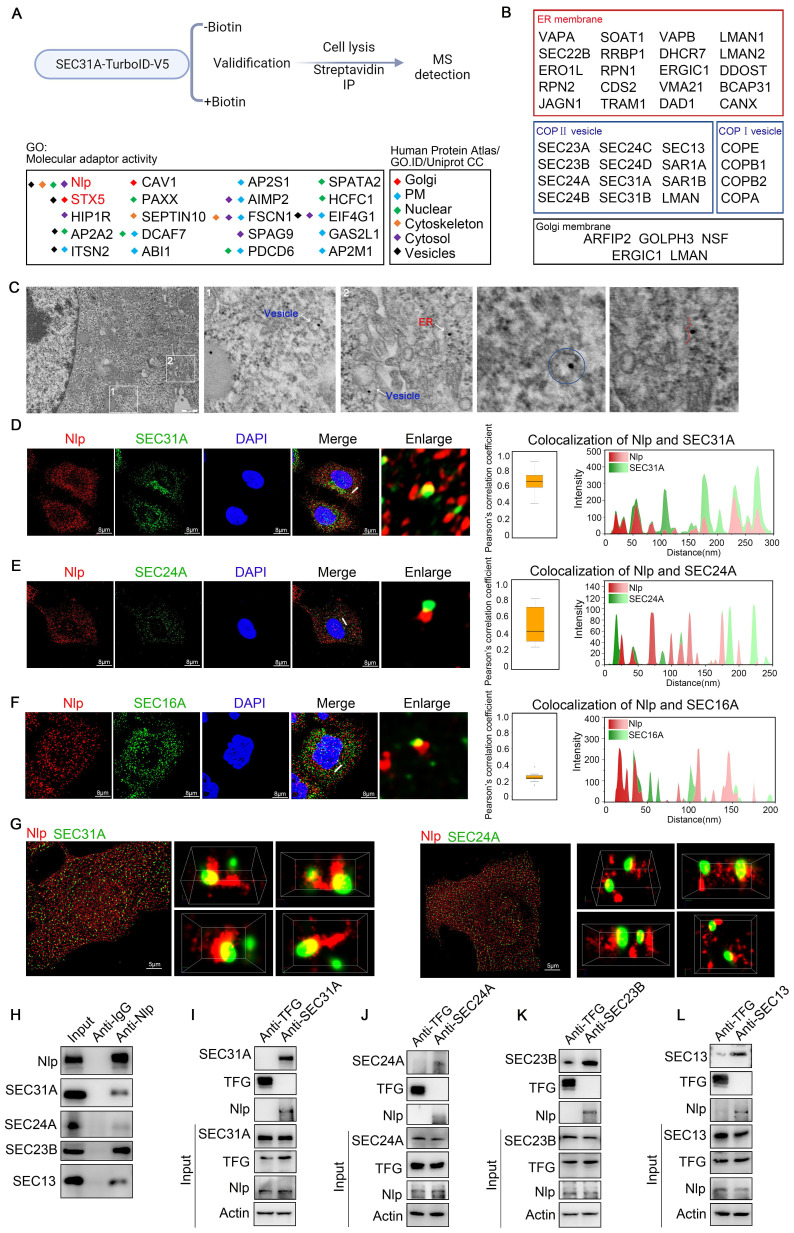
** Nlp interacted with main components of COPII-coated vesicles.** (A) Experimental design for MS-based proteomics (top). SEC31A-TurboID-V5 samples were labeled with or without 50 μM biotin for 10 min. Cells were then lysed, and biotinylated proteins were enriched using streptavidin beads for LC-MS/MS. Proteins analyzed here were selected by FC≥4 and p<0.05 compared to without biotin negative control groups. Intracellular distributions of “Molecular adapter activity” in SEC31A-TurboID MS results (bottom). Localizations of all proteins were verified by Human Protein Atlas, GO. ID and Uniprot CC. (B) Distributions of Nlp-interacted proteins in ER membrane, COPII-coated vesicle membrane, COPI-coated vesicle membrane and Golgi membrane. (C) Immunogold TEM labeled by anti-Nlp in HeLa cells. (Scale bar, 500 nm.). (D-E) Colocalization of Nlp and (D) SEC31A, (E) SEC24A by LSM980. Pearson's correlation coefficient was analyzed (middle). Relative intensity profile of white line in Merge channel was indicated (right). (Pearson's correlation coefficient rate: 50 cells from 3 experiments. Scale bar, 8 μm.). (F) Colocalization of Nlp and SEC16A by SP8 DIVE. Pearson's correlation coefficient was analyzed (middle). Relative intensity profile of white line in Merge channel was indicated (right). (Pearson's correlation coefficient rate: 30 cells from 3 experiments. Scale bar, 8 μm.). (G) Two-color three-dimensional N-STORM images of Nlp and SEC31A (left) or SEC24A (right) localizations in the HeLa cells. (Scale bar, 5 μm.). (H) Co-IP assay using anti-Nlp or anti-IgG in HeLa cell lysis. (I-L) Co-IP assay using anti-TFG, anti-SEC31A, anti-SEC24A, anti-SEC23B or anti-SEC13 in HeLa cell lysis.

To gain a comprehensive view of candidate proteins that were associated with the specificity and continuity of ER-to-Golgi vesicles, we constructed a SEC31A-TurboID-V5 system for proximity protein labeling in HeLa cells. After validifying the system (Fig. S1, A-B), we performed three independent quantitative mass spectrometry (MS) experiments and identified SEC31A associated proteins, and the control groups of which were set as “without biotin” group (n=3, p<0.05, and FC≥4). As recent studies have shown that activating adapters acted as dynein-dynactin complexes stability maintainers or switches during cargo trafficking through microtubules 31, we focused on the “Molecular adapter activity” proteins from the MS results to identify the potential molecules that might function in ER-to-Golgi vesicle transport. After listing and classifying the top 20 adapters by their known localization, we identified vesicle related adapters: Nlp (rank first), STX5, HIP1R, AP2A2 and ITSN2 (Fig. 1A). Among the adapters, HIP1R, AP2A2 and ITSN2 were localized in Clathrin-coated vesicles 39-41, and STX5 was already known as cargo transporter between ER and Golgi 28, while Nlp might be another potential adapter of ER-to-Golgi vesicles. To explore the deeper insights into the adapter character of Nlp, we performed quantitative mass spectrometry and identified a total of 1180 proteins via two independent Co-IP experiments using two different Nlp antibodies. Based on GO Enrichment Analysis, Nlp-interacting proteins were enriched in vesicle or protein mediated transport, notably including “vesicle-mediated transport” and “ER-to-Golgi vesicle-mediated transport” (Fig. S1C). Furthermore, the component distributions of Nlp-interacted proteins were enriched in ER membrane, COPII-coated vesicle membrane (GO:0030134, homo species, 21/94), COPI-coated vesicle membrane (GO: 0030663, homo species, 4/28) and Golgi membrane, including outer COPII layer SEC31/SEC13, inner COPII layer SEC23/SEC24, main ERES component SEC16A and small GTP-binding protein (GTPase) SAR1, suggesting that Nlp might be involved in the communications between ER and Golgi (Fig. 1B and Fig. S1D).

We next employed immune electron microscopy and confirmed that Nlp indeed distributed around ER and intracellular vesicles (Fig. 1C). To further identify whether Nlp was localized to COPII-coated vesicles, we carried out confocal assay between Nlp and COPII subunits in HeLa cells using LSM980. We used the colocalization of SEC31A and EEA1 as a control 42, 43. The results showed that Nlp was colocalized with partial outer COPII key component SEC31A and inner COPII key component SEC24A in an intersecting mode, and the Pearson's correlation coefficient rates were about 0.7 and 0.4 respectively (Fig. 1, D-E and Fig. S1F). Since SEC16A was the main component of COPII-coated vesicle budding site, we observed that Nlp was able to colocalize with SEC16A (Fig. 1F). These data indicated Nlp was colocalized with partial COPII-coated vesicles in ERESs. To further confirm the spatial colocalization between Nlp and COPII-coated vesicles, we performed super-resolution microscopy N-STORM that provided lateral resolution up to 20 nm (Fig. 1G and Movie S1-2). Consistent with the previous results, three-dimensional structure displays of intermolecular position relation between Nlp and SEC31A showed that SEC31A was colocalized with Nlp in an intersecting or adjacent mode.

In some cases, Nlp even grabbed SEC31A like a hand. Furthermore, three-dimensional maps also indicated that the localization between Nlp and SEC24A mostly appeared in an adjacent mode compared to SEC31A.

To further confirm the relationship between Nlp and COPII-coated vesicle, we performed Co-IP assays using anti-Nlp antibody or anti-IgG antibody in HeLa cell lysis. The results showed that SEC31A, SEC24A, SEC23B and SEC13 were co-immunoprecipitated by anti-Nlp antibody in Co-IP assays (Fig. 1H). Furthermore, previous researches demonstrated that Trk-fused gene (TFG) could compete with SEC31 for SEC23 binding to uncoating the coats of COPII-coated vesicles 44. Accordingly, we explored the relationship between Nlp and TFG by Co-IP assay, and the result indicated that Nlp was unable to interact with TFG (Fig. S1E). Thus, we targeted TFG as a negative control and performed Co-IP assay using anti-TFG antibody and anti-SEC31A antibody, anti-SEC24A antibody, anti-SEC23B antibody, or anti-SEC13 antibody respectively. Results showed that Nlp was co-immunoprecipitated by anti-SEC31A antibody, anti-SEC24A antibody, anti-SEC23B antibody, and anti-SEC13 antibody in Co-IP assays (Fig. 1, I-L).

When we investigated the relative locations between Nlp and COPI-coated vesicle membrane components using LSM980 in stable HeLa-EGFP-Nlp cells, we found that Nlp could colocalize with partial COPA, COPB, COPD and COPG in an intersecting or tangential manner (Fig. S1, G-J). These results suggested that Nlp might function as an adapter of ER-to-Golgi vesicle.

### Nlp altered assembling of COPII-coated vesicles as an adapter

**Figure 2:**
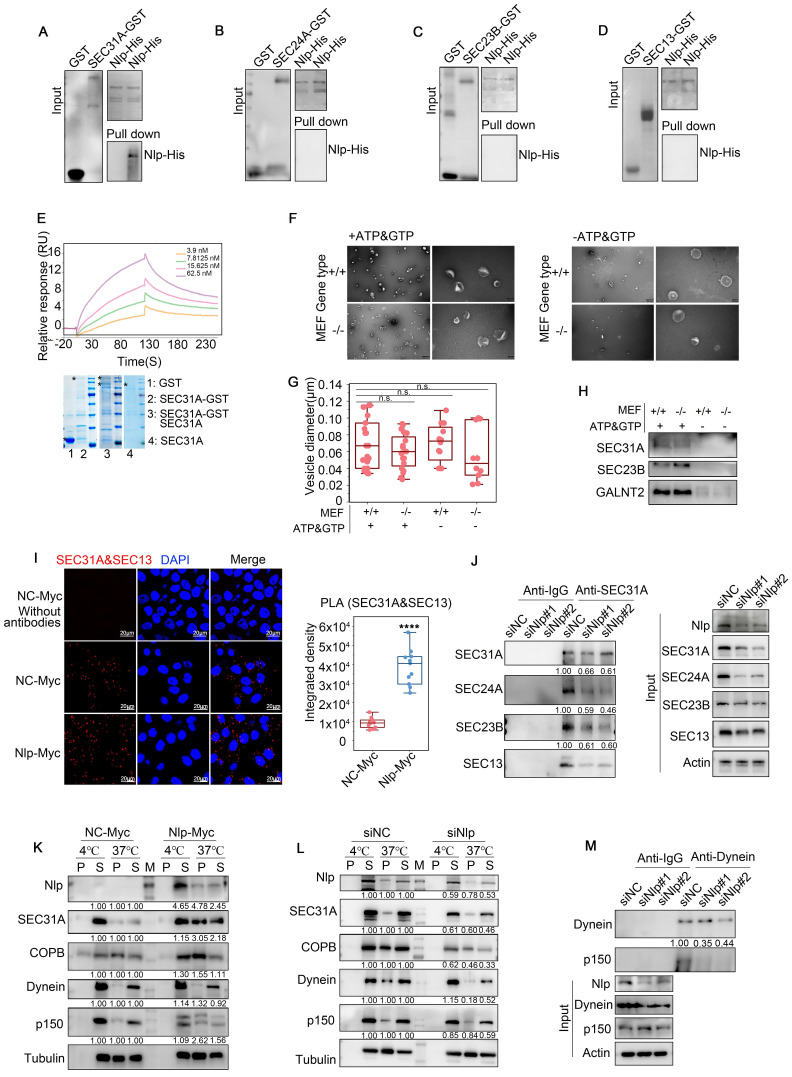
** Nlp altered assembling and stability of ER-to-Golgi vesicles through binding with SEC31A directly.** (A-D) GST pull-down assay using Nlp-His purified protein and GST or (A) SEC31A-GST, (B) SEC24A-GST, (C) SEC23B-GST or (D) SEC13-GST purified protein respectively. (E) SPR using Nlp-His purified protein and different concentrations of SEC31A purified protein. (F) Negative staining TEM visualization of the morphology of the vesicle membrane structures formed by *in vitro* vesicle formation assay. Membrane source was from MEF-*Nlp^-/-^* or MEF-*Nlp^+/+^* cells, and cytosolic protein source was HeLa cell lysis. (Scale bar, 500nm and 100nm.). (G) Vesicle diameter of (F). (10-20 vesicles from 2 experiments. n.s. represented no significance.). (H) Immunoblotting of vesicles from *in vitro* vesicle formation. (I) PLA signals of SEC31A and SEC13 in HeLa cells. (****p<0.0001. Scale bar, 20 μm.). (J) Co-IP assay using anti-SEC31A or anti-IgG in HeLa cells that have been transfected with siRNA for 48 hours. (K-L) Microtubule co-sedimentation assays of HeLa cells. The pellet (P) of 37°C incubation represented the microtubule-binding fraction, and supernatant (S) represented the unbound fraction. 4°C incubation acted as control. (M) represented Marker. (M) Co-IP assay using anti-Dynein or anti-IgG in HeLa cells that have been transfected with siRNA for 48 hours.

As Nlp could interact with ER-to-Golgi vesicles, we next investigated whether this interaction occurred in a direct manner. We expressed and purified both full-length SEC31A-GST and Nlp-6*His proteins from *E. coli*, and performed GST pull-down assay. Results suggested that purified SEC31A-GST directly interacted with purified Nlp-6*His, while SEC24A-GST, SEC23B-GST and SEC13-GST could not bind with Nlp-6*His (Fig. 2, A-D and Fig. S1K). In addition, SEC31A purified protein was obtained from SEC31A-GST purified protein using Thrombin, and then SPR assay was used to further verify the interaction between SEC31A and Nlp-6*His purified proteins. Results showed that purified SEC31A bound with Nlp-6*His directly in a medium intensity (Fig. 2E). To map the binding region between Nlp and SEC31A, six truncated constructs of Nlp were made according to its functional domains (Fig. S1L). Firstly, SEC31A purified protein was obtained from SEC31A-GST purified protein using Thrombin. After verifications by Coomassie brilliant blue, silver staining and immunoblotting, GST pull-down assay was performed using GST-tag purified proteins of Nlp truncates and SEC31A purified protein. These data indicated that Nlp-CC2 domain interacted with SEC31A directly (Fig. S1M).

We next explored whether Nlp affected the assembly of ER-to-Golgi vesicles through *in vitro* vesicle formation assay, as well as *in vivo* PLA and Co-IP assay. Briefly, for vesicle formation assay, we used mouse embryonic fibroblast of experimental groups MEF-*Nlp^-/-^* or wild type control groups MEF-*Nlp^+/+^* as the donor membranes of reconstituted vesicles after digitonin-permeabilized, and utilized HeLa cell lysis or HEK293T cell lysis as the cargoes of newly assembled vesicles according to protocol reported previously 45. After collecting the donor membranes, membranes and cell lysis were incubated with or without GTP and ATP regeneration system to reconstruct vesicles *in vitro*. Then we collected the vesicles by Opti-Prep, and analyzed by negative stain electron microscope as well as immunoblotting. After exploring the components and sizes of vesicles through immunoblotting and quantification of the vesicles' diameters, we verified that the vesicles we obtained contained COPII-coated vesicles. Then we observed the shapes of the vesicles, and found that compared to MEF-*Nlp^+/+^* group, deficiency of Nlp greatly affected the assembly of vesicles (Fig. 2, F-H and Fig. S2A). Therefore, we focused on the mechanism how Nlp affected the assembly of COPII-coated vesicles. *In vivo* PLA showed that the proximity between SEC31A and SEC13 was increased in Nlp overexpressed cells compared with that seen in the control group (Fig. 2I). In contrast, SEC31A bound less SEC24A/SEC23B/SEC13 when Nlp was knocked down in HeLa cells (Fig. 2J). In addition, compared to the control group, the protein levels of main COPII-coated vesicle layer components of HeLa cells were down-regulated following Nlp knockdown, while all these proteins were up-regulated in HeLa-EGFP-Nlp cells (Fig. S2, B-C). These findings suggested that the adapter role of Nlp could enhance the stability of COPII-coated vesicle coats through acting as a platform.

Nlp is essential for the proper assembly and activation of the dynein motor complex, and could enhance motile properties of dynein/dynactin in the presence of LIS1^32^, ^33^, ^46^. Meanwhile, Nlp participated in COPII-coated vesicle coats formation through interacting with SEC31A directly, so we further investigated the mechanism of adapter character of Nlp through microtubule co-sedimentation assays (Fig. 2, K-L). In this experiment, we reconstructed microtubules from HeLa cell lysis in condition of 37°C or 4°C respectively. The results indicated that Nlp indeed played a role in ER-derived vesicle trafficking as an adapter. Consistent with this observation, the results in Co-IP assay further confirmed that Nlp was crucial in maintenance of dynein-dynactin complexes (p150 was the largest subunit of dynactin) (Fig. 2M). Collectively, we demonstrated that Nlp played significant roles as an adapter in ER-derived vesicle.

### Nlp had cargo specificity of COPII-coated vesicles

**Figure 3:**
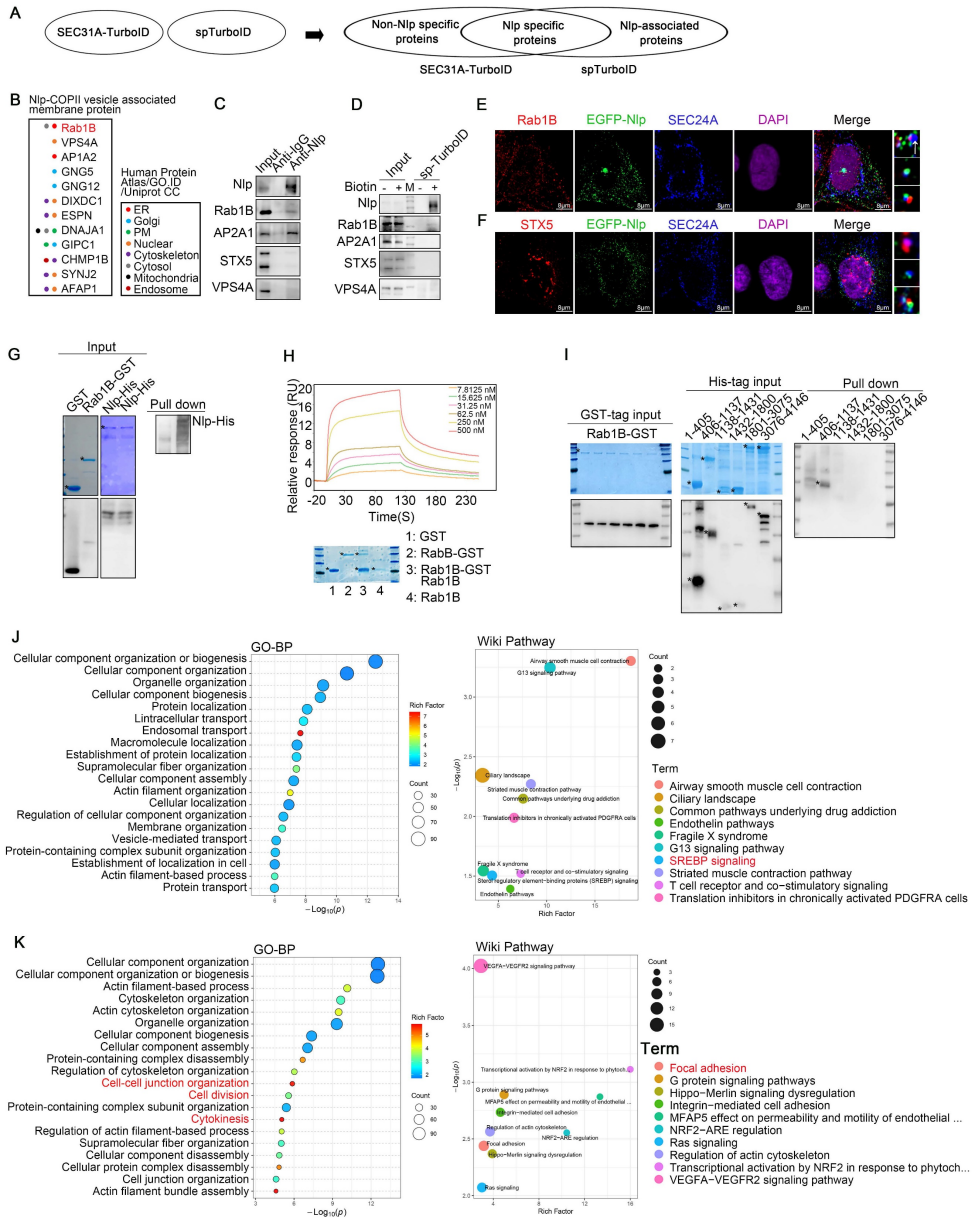
** Nlp cooperated with Rab1B to participate in specific cargoes transport.** (A) Experimental design for MS-based proteomics. Split-TurboID (spTurboID) samples were labeled without Biotin, with 50 μM biotin or with 50 μM biotin & 100 nM rapamycin for 4 hours. Cells were then lysed, and biotinylated proteins were enriched using streptavidin beads for LC-MS/MS. Proteins analyzed here were selected by FC≥4 and p<0.05 compared to negative control groups. (B) Intracellular distributions of cargo transport related membrane proteins from “Membrane proteins” in split-TurboID MS results. All proteins were verified by Human Protein Atlas, GO. ID and Uniprot CC. (C) Co-IP assay using anti-Nlp or anti-IgG in HeLa cell lysis. (D) Immunoblotting of HeLa cells that have been transfected with Split-TurboID system plasmids for 36 hours. Cells were labeled with or without 50 μM biotin for 4 hours before washing and collecting for streptavidin-IP. (E) Colocalization of EGFP-Nlp, SEC24A and Rab1B by LSM980. The interaction ratio of Rab1B and SEC24A was about 38.8%. The interaction ratio of only EGFP-Nlp and SEC24A was about 10.8%. The interaction ratio of total EGFP-Nlp and SEC24A was about 23%. The interaction ratio of EGFP-Nlp, Rab1B and SEC24A was about 12.2%. (Scale bar, 8 μm.). (F) Colocalization of EGFP-Nlp, SEC24A and STX5 by LSM980. The interaction ratio of STX5 and SEC24A was about 22.8%. The interaction ratio of EGFP-Nlp and SEC24A was about 21.1%. The interaction ratio of EGFP-Nlp, Rab1B and SEC24A was about 0.02%. (Scale bar, 8 μm.). (G) GST pull-down assay using Nlp-His purified protein and GST or Rab1B-GST purified protein respectively. (H) SPR using Nlp-His purified protein and different concentrations of Rab1B purified protein. (I) After verifications by Coomassie brilliant blue and Western blot, GST pull-down assay was performed using His-tag purified proteins of Nlp truncates and Rab1B-GST purified protein. (J-K) Results of SEC31A-TurboID and Split-TurboID were grouped into three parts: the intersection of the TurboID and split-TurboID as Nlp-specific sorting/transporting proteins; the remaining part of TurboID as non-Nlp-specific sorting/transporting proteins; the remaining part of split-TurboID as Nlp-associated proteins. Figures represented enrichment analysis of Biology Process and WiKi Pathway of (J) non-Nlp specific sorting/transporting proteins, (K) Nlp specific sorting/transporting proteins.

Since Nlp could function as an adapter during ER-to-Golgi vesicles formation, and was colocalized with partial COPII-coated vesicles as well as ERESs, we hypothesized that Nlp might localize to specific ERES to collect particular cargoes or transport specific COPII-coated vesicles. Thus, we analyzed whether Nlp influenced the specificity of cargoes by TurboID and split-TurboID experiments 47. We designed SEC31A-FRB-split-TurboID(C) and Nlp-FKBP-split-TurboID(N) systems to capture COPII-coated vesicle cargoes that might be specifically sorted or transported by Nlp in HeLa cells (Fig. 3A). After immunoblotting, silver staining assay and confocal verification (Fig. S2, D-E), we analyzed the specificity of proteins by mass spectrometry (MS). We used “without biotin & rapamycin” (n=3) as control group for split-TurboID system, and we took the overlapping parts of “with biotin” (n=3) and “with biotin & rapamycin” (n=3) groups for analysis. In addition, we applied data of TurboID system from Figure 1 as whole potential COPII-coated vesicle cargoes.

Firstly, to find the membrane protein that might cooperate with Nlp-dynein-dynactin system to keep the continuity of cargoes during COPII-COPI coats transformation and take part in the directionality of cargoes during transportation from ER-to-Golgi, we analyzed MS results of split-TurboID system (Fig. 3B). After analyzing “Nlp-COPII vesicle associated membrane proteins” and verifying the potential proteins by Co-IP, confocal, as well as streptavidin-IP by split-TurboID system, we found Rab1B as one of Nlp associated peripheral membrane proteins. Besides, overexpression of Nlp changed the distribution of Rab1B (Fig. S2F). On account of STX5 was known as ER-to-Golgi partial cargoes (such as pro-collagens and low-density lipoproteins) selector and transporter 28, 29, and Nlp bound with partial ER-to-Golgi vesicles, we further explored the specificity of these adapters during vesicle transportation. Interestingly, our confocal results showed that Nlp and STX5 were colocalized with different COPII-coated vesicles, which meant adapters in different vesicles formation sites might have their uniqueness. In rare circumstances, Nlp might cooperate with STX5 (Fig. 3, C-F).

Firstly, to further explore the relationship between Nlp and Rab1B, we expressed and purified both full-length Rab1B-GST and Nlp-6*His proteins from *E. coli*, and performed GST pull-down assay. Results suggested that purified Rab1B-GST directly interacted with purified Nlp-6*His (Fig. 3G). In addition, Rab1B purified protein was obtained from Rab1B-GST purified protein using Thrombin, and then SPR assay was used to further verify the association between Rab1B and Nlp-6*His purified protein. Results showed that purified Rab1B bound with Nlp-6*His directly in a medium intensity (Fig. 3H). To determine the binding region between Nlp and Rab1B, GST-tag and 6*His-tag proteins of truncated constructs of Nlp were purified according to its functional domains (Fig. S1L). Firstly, GST pull-down assay was performed using His-tag purified proteins of Nlp truncates and Rab1B-GST purified protein, and the data indicated that Nlp-EF34 domain interacted with Rab1B directly (Fig. 3I). Moreover, Rab1B purified protein was obtained from Rab1B-GST purified protein using Thrombin. After verifications by Coomassie brilliant blue and immunoblotting, GST pull-down assay was performed using GST-tag purified proteins of Nlp truncates and Rab1B purified protein. These data indicated that Nlp-EF34 domain interacted with Rab1B directly (Fig. S2G). Therefore, these data demonstrated that adapter Nlp cooperated with Rab1B to participate in ER-derived vesicles trafficking.

Then we divided the MS results into three groups for GO analysis, Wiki pathway analysis or KEGG pathway analysis: the intersection of the TurboID and split-TurboID system as Nlp-specific sorting/transporting proteins; the remaining part of TurboID system as non-Nlp-specific sorting/transporting proteins; the remaining part of split-TurboID system as Nlp-associated proteins (Fig. 3A). These results preliminarily provided important information on Nlp-specific sorting/transporting proteins, including cytokinesis related proteins, focal adhesion related proteins, G proteins, RNA binding proteins and other proteins. Most of these proteins did correspond to Nlp functions or domains previously reported 48-50.

**Figure 4:**
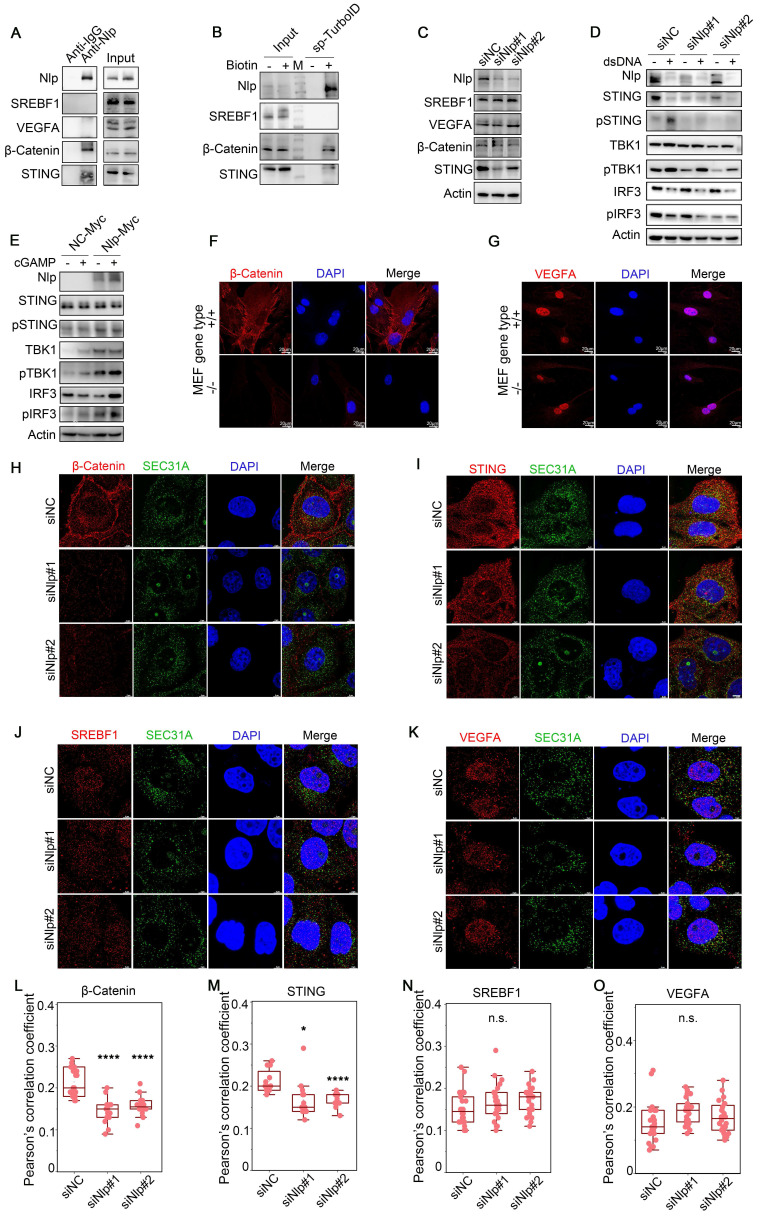
** Nlp-dependent specific cargoes verification.** (A) Co-IP assay using anti-Nlp or anti-IgG in HeLa cell lysis. (B) Immunoblotting of HeLa cells that have been transfected with Split-TurboID system plasmids for 36 hours. Cells were labeled with or without 50 μM biotin for 4 hours before washing and collecting for streptavidin-IP. (C) Immunoblotting of Hela cells that have been transfected with siRNA for 48 hours. (D) HeLa cells were transfected with siRNA for 48 hours. Then cells were treated with or without 2μg dsDNA for 2 hours before immunoblotting. (E) HeLa cells were transfected with plasmids for 36 hours. Then cells were treated with or without 0.5 μM cGAMP using 10 μg/mL digitonin for 15 min. After treated cells with cGAMP for 2 hours, cells were collected for immunoblotting. (F-G) Confocal of (F) β-Catenin or (G) VEGFA in MEF-*Nlp^-/-^* and MEF-*Nlp^+/+^* cells using Leica. (Scale bar, 20 μm.). (H-K) Colocalization of SEC31A and (H) β-Catenin, (I) STING, (J) SREBF1 or (K) VEGFA in HeLa cells that have been transfected with siRNA for 48 hours using Leica. (Scale bar, 5 μm.). (L-O) Pearson's correlation coefficient of (H-K). (Pearson's correlation coefficient rate: 20-30 cells from 2 experiments. n.s. represented no significance, *p<0.05, ****p<0.0001.).

While the non-Nlp-specific sorting/transporting proteins mainly gathered in SREBP signaling pathway, and the Nlp-associated proteins gathered in ER stress pathway (Fig. 3, J-K, and Fig. S2, H-J). Therefore, we verified some proteins shown by MS results through Co-IP and streptavidin-IP, including β-Catenin, STING, SREBF1 and VEGFA. All these proteins were transported through ER-to-Golgi vesicles 4, 51-53. Results showed that Nlp might sort and transport β-Catenin and STING, while not interact with SREBF1 and VEGFA (Fig. 4, A-B). In addition, the protein levels of β-Catenin and STING were down-regulated in different degrees when Nlp was knocked down by siRNA, while the protein levels of SREBF1 and VEGFA were not affected (Fig. 4C).

To further investigate the adapter role of Nlp in specific cargo transporting, we explored the relationship between Nlp and cGAS-STING signaling pathway, the activation of which depended on ER-to-Golgi vesicle transport. Firstly, HeLa cells were treated with or without 2μg dsDNA for 2 hours to induce cGAS-STING pathway, and we found that the down-regulation of Nlp decreased STING-TBK1-IRF3 axis (Fig. 4D). Then we treated HeLa cells with or without 0.5 μM cGAMP for 2 hours as previously reported 4, and the data suggested that higher expression level of Nlp increased STING-TBK1-IRF3 axis (Fig. 4E). Meanwhile, we investigated whether Nlp influenced the distribution of β-Catenin or VEGFA in MEF-*Nlp*^-/-^ and the wild type MEF-*Nlp*^+/+^ cells (Fig. 4, F-G). Results showed that the distribution of β-Catenin in cell membrane decreased dramatically when Nlp was absent, while the distribution of VEGFA was not affected by the Nlp. Furthermore, the colocalization between SEC31A and β-Catenin or STING was decreased when Nlp was knocked down in HeLa cells, while the colocalization between SEC31A and SREBF1 or VEGFA was not affected (Fig. 4, H-O).

Collectively, these results indicated Nlp not only offered a platform for the formation of vesicles in ERESs, but took part in specific cargoes selection or transportation through cooperating with Rab1B directly.

### Nlp regulated ER-to-Golgi vesicles localization and transformation

**Figure 5:**
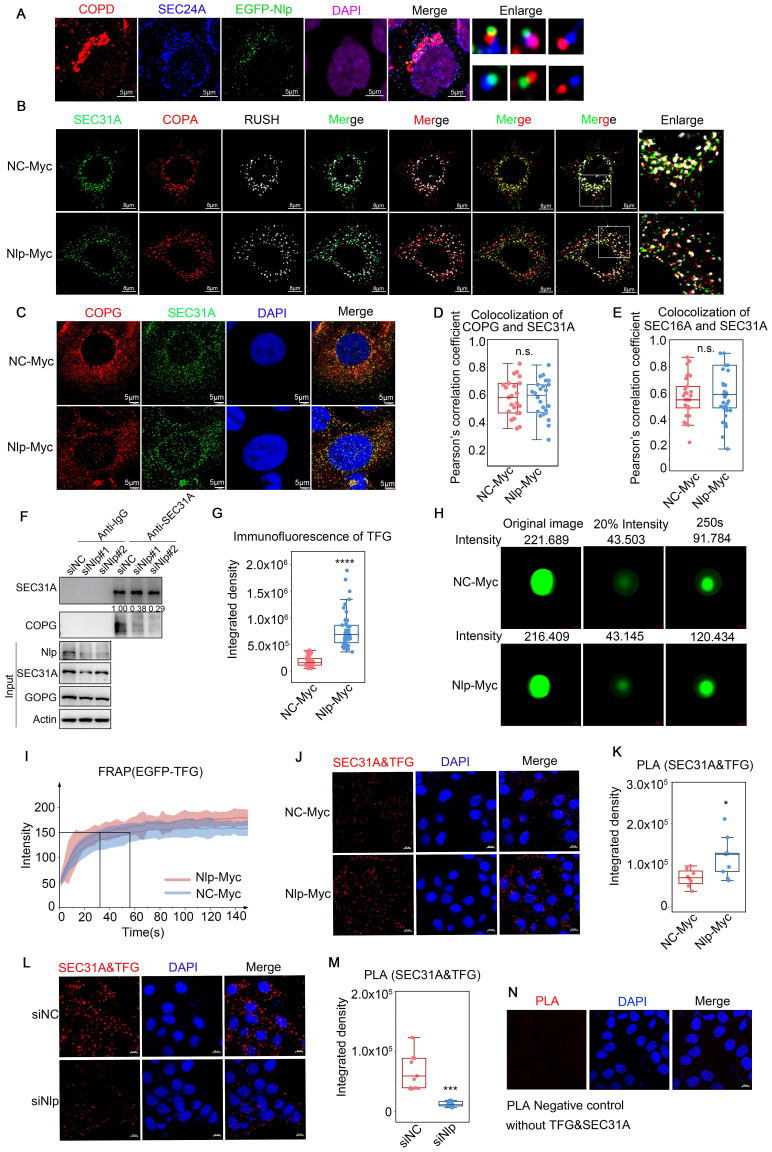
** Nlp regulated transformation and uncoating of ER-to-Golgi vesicles.** (A) Colocalization of EGFP-Nlp, SEC24A and COPD by LSM980 in HeLa cells. (Scale bar, 5 μm.). (B) Colocalization of SEC31A, COPA and RUSH by LSM880 in HeLa cells that have been transfected with plasmids for 36 hours. (Scale bar, 8 μm.). (C) Colocalization of COPG and SEC31A by Leica in HeLa cells that have been transfected with plasmids for 36 hours. (Scale bar, 5 μm.). (D) Pearson's correlation coefficient of (C). (Pearson's correlation coefficient rate: 20-30 cells from 2 experiments. n.s. represented no significance.). (E) Colocalization analysis of SEC31A and SEC16A by LSM780 in HeLa cells that have been transfected with plasmids for 48 hours. Confocal figures were presented in Figure S3A. (Pearson's correlation coefficient rate: 30 cells from 3 experiments. n.s. represented no significance.). (F) Co-IP assay using anti-SEC31A or anti-IgG in HeLa cells that have been transfected with siRNA for 48 hours. (G) Confocal assay represented the aggregation ability of TFG in HeLa cells that have been transfected with plasmids for 48 hours. Confocal figures were presented in Figure S3E. (****p<0.0001.). (H) FRAP of EGFP-TFG in HeLa cells that have been transfected with plasmids for 36 hours. The intensity of EGFP-TFG was decreased to 20% using photobleaching and observed its recovery ability for 150s. (I) FRAP analysis of experiment described in (H). Mean and standard deviation of 15 puncta each group were indicated. (J-N) PLA signals of SEC31A and TFG in HeLa cells that have been transfected with plasmids or siRNA for 48 hours. Negative control was shown in (N). (Scale bar, 10 μm. *p<0.05, ***p<0.001.).

**Figure 6:**
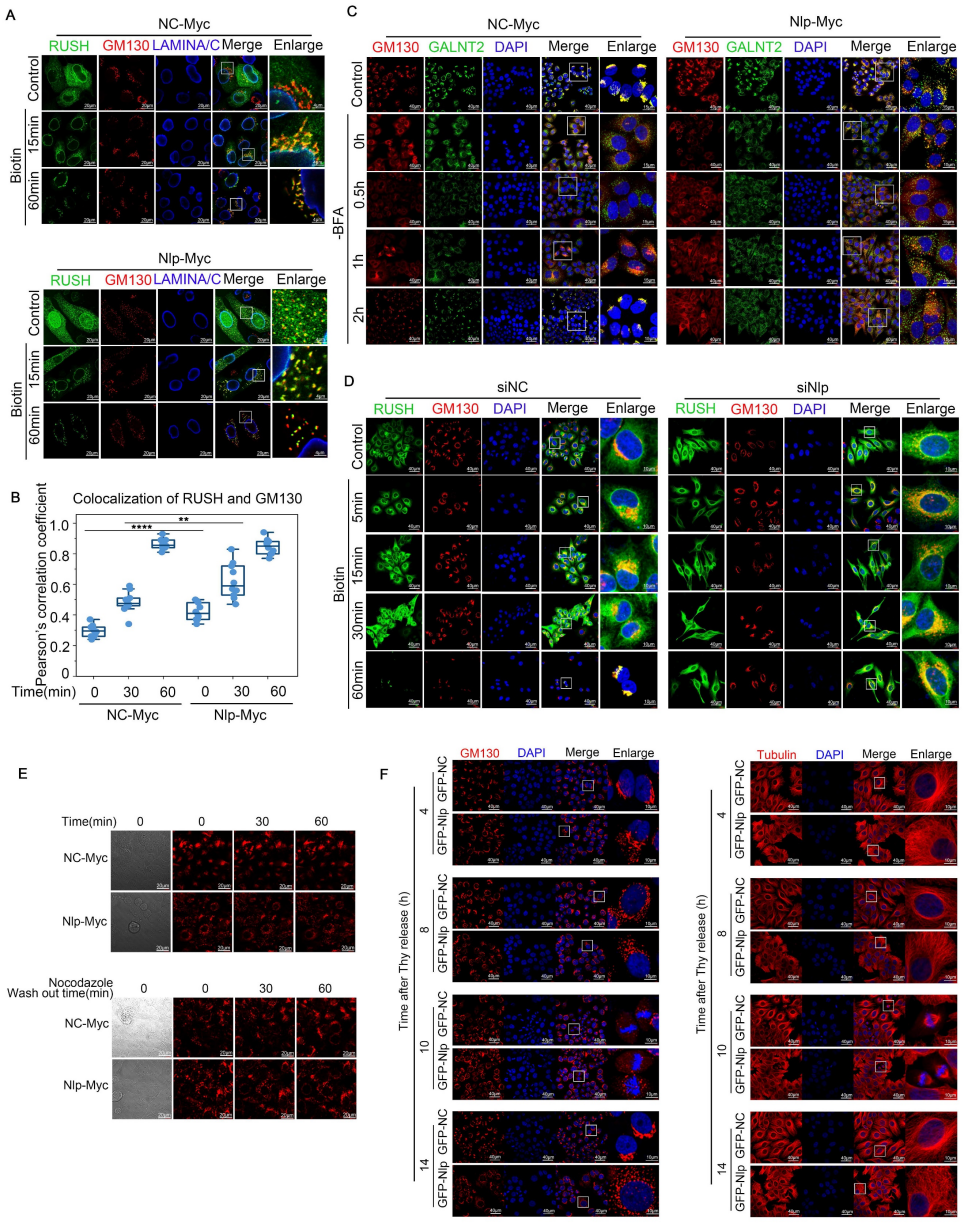
** Alteration of Nlp induced remodeling of microtubules and changed ER-to-Golgi vesicle trafficking manner.** (A) Confocal for RUSH assay. HeLa cells were transfected with plasmids for 36 hours, and then incubated with 80 μM of biotin for different time (0-60 min) for confocal assay. (Scale bar, 20 μm or 4 μm.). (B) Pearson's correlation coefficient of (A). (Pearson's correlation coefficient rate: 15-30 cells from 2 experiments. **p<0.01, ****p<0.0001.). (C) Colocalization of GALNT2 and GM130 by LSM780 in HeLa cells that have been transfected with Nlp-Myc or NC-Myc for 36 hours. After the transfection, cells were treated with 5 μg/mL BFA for 30 min, and removed BFA for different times to capture the movements of vesicles. (Scale bar, 40 μm or 15 μm.). (D) Confocal for RUSH assay. HeLa cells were transfected with plasmids and siRNA for 36 hours, and then incubated with 80 μM of biotin for different time (0~60 min) for confocal assay. (Scale bar, 40 μm or 10 μm.). (E) HeLa cells were treated with or without 5 μg/mL Nocodazole for 2 hours, and then changed the Nocodazole containing media with fresh DMEM for time lapse experiment. (Scale bar, 20 μm). (F) Stable control HeLa-C3 or HeLa-EGFP-Nlp cells were arrested in G1-S boundary and released into fresh medium. Cells were collected every 4 hours for confocal. (Scale bar, 40 μm or 10 μm.).

COPII-coated vesicles formed and budded in ERESs and afterwards COPII-coated vesicle coats would disassemble around ERES pocket or ERGIC to transformed to COPI-coated vesicles 15, 21. As our observations suggested that Nlp could not only act as a platform for the assembly of COPII-coated vesicle, but also interact with COPI-coated vesicle as an adapter, we continued to identify the role of Nlp in transformation of ER-to-Golgi vesicles. Firstly, we analyzed the relationships between EGFP-Nlp, COPII-coated vesicles and COPI-coated vesicles in stable HeLa-EFGP-Nlp cells, and found that EGFP-Nlp was colocalized with both COPII and COPI-coated vesicles in different manners: EGFP-Nlp colocalized with COPII or COPI-coated vesicles independently, or colocalized with both COPII and COPI-coated vesicles (Fig. 5A). Then we applied RUSH system (adding biotin for 3 minutes, the RUSH system was validified in Fig. 6 and Movie S4-5) or SEC16A as ERESs, and performed confocal assay in HeLa cells. In the RUSH assay, HeLa cells were transfected with Str-KDEL-SBP-GFP plasmid. Str-KDEL is an ER retention signal that retained the signals at the ER like a hook, while ST-SBP is a Golgi reporter. Addition of biotin can release the reporter from ER synchronously and localize to Golgi. Results showed that overexpression of Nlp dispersed the distribution of ERESs, COPII-coated vesicles, and COPI-coated vesicles, without changing the colocalization between ERESs and ER-to-Golgi vesicles (Fig. 5, B-E, and Fig. S3A). Furthermore, the re-distributions of Nlp and SEC31A were consistent when Nlp was knocked down in HeLa cells using mixture of two Nlp siRNA (Fig. S3, B-C). Interestingly, when Nlp was knocked down in HeLa cells, the interaction between COPII and COPI-coated vesicles was down-regulated (Fig. 5F). These results indicated that Nlp participated in the localization and transformation of COPII and COPI-coated vesicles.

It has been found that TFG bound to SEC23 in a concentration-dependent aggregation manner and promotes outer coat dissociation ultimately 44, 54-56. Although Nlp could not interact with TFG or affect the binding ability between SEC23B and TFG (Fig. S1E and Fig. S3D), we found that the aggregation ability of TFG was enhanced upon Nlp overexpression (Fig. 5G and Fig. S3E). Therefore, we continued to explore the relationship between Nlp and TFG. Firstly, we applied FRAP assay to detected whether Nlp influenced the aggregation ability of EGFP-TFG in HeLa cells.

The intensity of EGFP-TFG was decreased to 20% using photobleaching and observed its recovery ability for 150s. As expected, the results indicated that the ability of TFG aggregation was up-regulated by overexpression of Nlp through facilitating the recovering time of TFG liquid drop significantly (Fig. 5, H-I, and Movie S3). In addition, the aggregated TFG gathered around COPII-coated vesicles were increased when the expression level of Nlp was up-regulated (Fig. 5, J-K and Fig. S3F). When we knocked down Nlp using mixture of two Nlp siRNA, TFG was diffused in the cytoplasm, and the colocalization of TFG and COPII-coated vesicles was disrupted (Fig. 5, L-M and Fig. S3G). Negative control without primary antibodies for PLA was shown in Figure 5N. Since Nlp could enhance cell stiffness 49 but not involved in the recruitment of TFG, we speculated that Nlp accelerated the process of COPII-coated vesicles uncoating by strengthening cell rigidity, which further enhanced the aggregation capacity of TFG around vesicles.

### Nlp influenced transportation of ER-to-Golgi vesicles

Previous studies have shown that COPII-coated vesicles were transformed to COPI-coated vesicles and then transported along microtubules in a dynein-dynactin complex depended manner 15. Thus, we further explored whether Nlp would influence the trafficking of ER-to-Golgi vesicles. Firstly, we investigated the distribution of Nlp besides vesicles or ER, and we found that Nlp could localize to cell membrane. Meanwhile, the trace of Nlp showed that after interacting with Golgi, some of Nlp particles moved towards periphery of cells, while others stayed longer at Golgi (Fig. S3, H-I). These data were consistent with the results that Nlp took part in transportation of β-Catenin.

To characterize how Nlp functioned in transporting of ER-to-Golgi vesicles, we utilized RUSH system and GalNAc-T2 transporting system. In brief, GalNAc-T2 is redistributed to ER in the presence of brefeldin A (BFA) and returns to Golgi apparatus upon BFA removal 57. When Nlp was overexpressed in RUSH system as well as GalNAc-T2 transporting system, COPII-coated vesicles were colocalized with the Golgi apparatus at an early stage, and our time lapse video showed that the vesicles mainly distributed around the site of cell movement (Fig. 6, A-C, Fig. S4, A-C and Movie S4). By contrast, RUSH signals would be trapped in ER when Nlp was knocked down (Fig. 6D and Movie S5). Additionally, Golgi was fragmentized in both Nlp knockdown and overexpression situations although the Golgi morphology was more fragmented in the low-expression group than the over-expression group. Since Nlp was a significant microtubule associated protein 33, we next investigated whether the fragmentation of Golgi caused by overexpression of Nlp was related to microtubules. We treated HeLa cells with or without 5 μg/mL Nocodazole for 2 hours, and then changed the Nocodazole containing media with fresh DMEM and took time lapse experiment for an hour. The results suggested that Golgi fragmentation caused by overexpression of Nlp was associated with microtubules (Fig. 6E). Then we explored the morphology of Golgi as well as microtubules under different protein levels of Nlp, and found that there was a correlation between distribution of microtubules and Golgi fragmentation when Nlp was overexpressed. Moreover, we excluded the cell cycle factor that might have influence on the morphology of microtubules and Golgi (Fig. 6F and Fig. S4D).

Since overexpression of Nlp led to fragmentation of Golgi, we continued to investigate whether the overexpression of Nlp generated functional changes of Golgi through testing purified Golgi apparatus by LC-MS in stable control HeLa-C3 and HeLa-EGFP-Nlp cells. After notarizing the morphology of Golgi in stable HeLa-C3 and HeLa-EGFP-Nlp cells, we purified the Golgi apparatus for LC-MS using Minute™ Golgi Apparatus Enrichment Kit (Fig. S5, A-C). Interestingly, compared to the Golgi of stable HeLa-C3 cells, microtubule cytoskeleton, microtubule organizing center and negative regulation of programmed cell death related components or processes were up-regulated in Nlp-overexpressed Golgi group, while ERGIC component and protein folding related processes were down-regulated (Fig. S5, D-F).

Taken together, these results demonstrated that Nlp was an activating adapter to trigger the transport of ER-to-Golgi vesicles along microtubules. Besides, through influencing the shapes of microtubules, as well as the distribution of vesicles, ERESs and Golgi, Nlp might reshape the morphology and distribution of organelles to improve the mode of organization in cells.

### Nlp altered protein homeostasis and reshaped ER-to-Golgi communication manner

**Figure 7:**
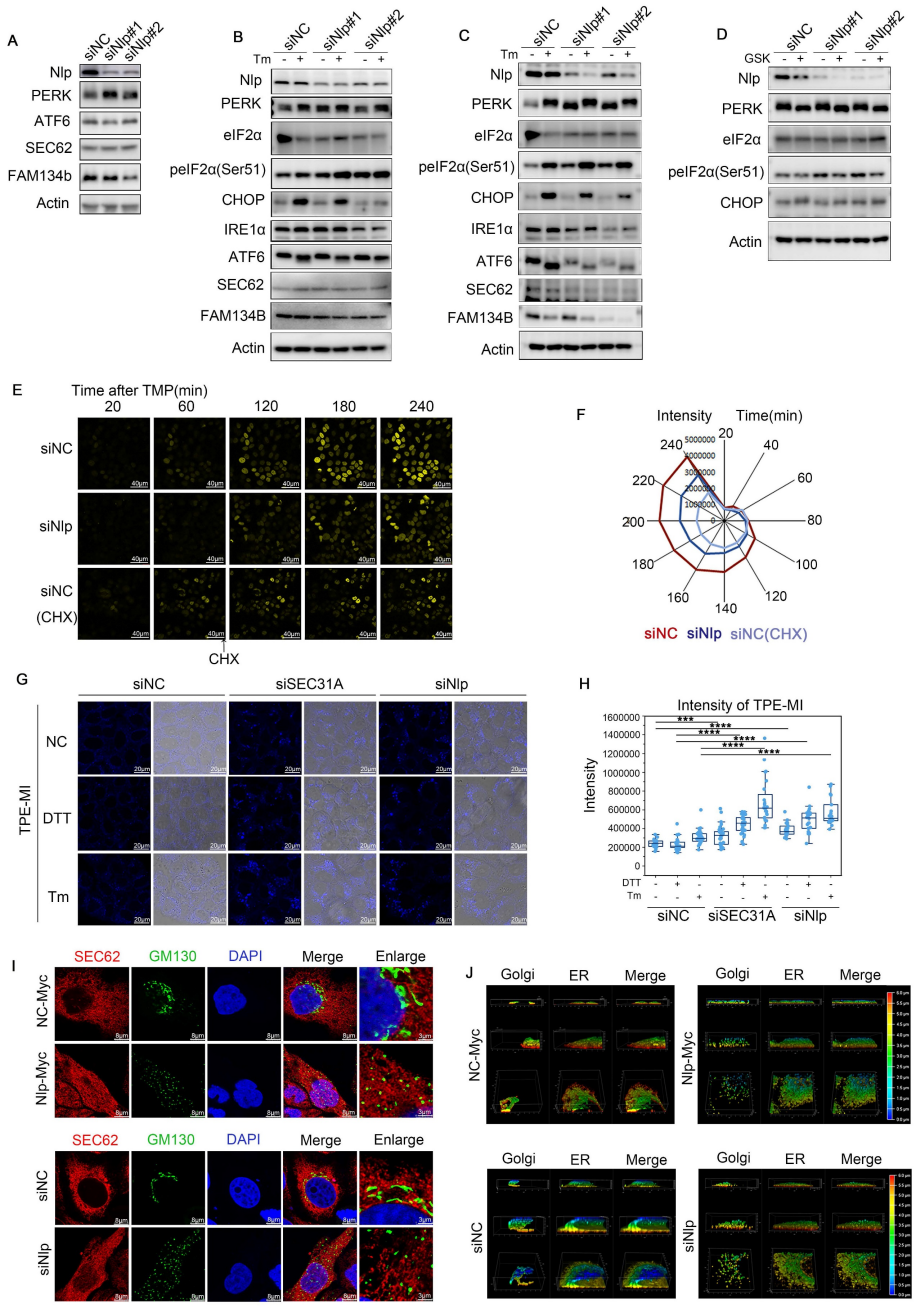
** Nlp caused ER stress and reshaped ER-Golgi communication manner.** (A) Immunoblotting of HeLa cells that have been transfected with siRNA for 48 hours. (B) Immunoblotting of HeLa cells that have been transfected with siRNA for 36 hours, and subsequently treated with tunicamycin (Tm) in a concentration of 0.5 μg/mL for 12 hours. (C) Immunoblotting of KYSE450 cells that have been transfected with siRNA for 36 hours, and subsequently treated with tunicamycin (Tm) in a concentration of 0.5 μg/mL for 12 hours. (D) Immunoblotting of KYSE450 cells that have been transfected with siRNA for 36 hours, and subsequently treated with GSK2606414 in a concentration of 6 μM for 12 hours. (E-F) Time lapse of stable TOP-H2B-YFP-DD HeLa cells that have been transfected with siRNA for 36 hours. (Scale bar, 40 μm.). (G-H) Confocal assay of TPE-MI, HeLa cells were transfected with siRNA for 48 hours before treated with 2.5 μg/mL Tm or 2 μM DTT for 2 hours. Then cells were treated with fresh 50 μM TPE-MI in PBS for 30 min at 37 °C before fixing. (Scale bar, 20 μm. ***p<0.001, ****p<0.0001.). (I) Confocal assay of Sec62 and GM130 by SP8 DIVE in HeLa cells that have been transfected with plasmids or siRNA for 48 hours. (Scale bar, 8 μm or 3 μm.). (J) Confocal assay of Sec62 (ER) and GM130 (Golgi) by SP8 STED in HeLa cells that have been transfected with plasmids or siRNA for 48 hours. Color represented depth.

Since lower expression of Nlp led to blockage of COPII-coated vesicles in ER, and vesicle budding failure led to ER stress 58, we next investigated whether the knockdown of Nlp caused ER stress. ER stress can induce UPR or ER-phagy to release the stress circumstances 59. Three main UPR pathways are activated when unfolded or misfolded proteins accumulate in the ER: ATF6 pathway, PERK pathway and IRE1α pathway. Among them, PERK pathway blocks mRNA translation on ribosome through phosphorylating eIF2α, otherwise PERK will activate CHOP to initiate apoptosis 60. Initially, we noticed the reduction of Nlp could up-regulate PERK pathway to relieve ER stress (Fig. 7A). Subsequently, ER stress activator tunicamycin (Tm) was applied in a concentration of 0.5 μg/mL for 12 hours to induce ER stress in HeLa cells and KYSE450 cells respectively, and the results showed that downregulation of Nlp could activate PERK pathway without the activation of CHOP or ER-phagy pathway (Fig. 7, B-C). Moreover, we utilized PERK inhibitor GSK2606414 in a concentration of 6 μM for 12 hours in KYSE450 cells, and observed that Nlp influenced PERK-eIF2α pathway (Fig. 7D). As the activation of PERK-eIF2α pathway can decrease the expression levels of most proteins, we introduced TOP-H2B-YFP-DD system 61 to further verify the roles of Nlp in protein translation. Consistently, knockdown of Nlp in stable TOP-H2B-YFP-DD HeLa cells down-regulated protein translation levels (Fig. 7, E-F). Therefore, we considered that downregulation of Nlp increased ER stress, and PERK-eIF2α pathway was activated to reduce the protein translation level and ease the stress condition. To confirm the direct or potential proteostasis and unfolded protein load under different Nlp protein levels, we applied Tetraphenylethene maleimide (TPE-MI) probe, the fluorescence of which would be activated upon free cysteine thiols 62. We prepared HeLa cells that transfected with siNC, siSEC31or siNlp for 48 hours, and treated with or without Tm or DTT for 2 hours before labelling with TPE-MI (Fig. 7, G-H). Coincidently, compared with the control group, unfolded protein loads of siSEC31A and siNlp were upregulated, and further increased when dealing with Tm or DTT. Taken all together, the downregulation of Nlp altered protein homeostasis.

**Figure 8:**
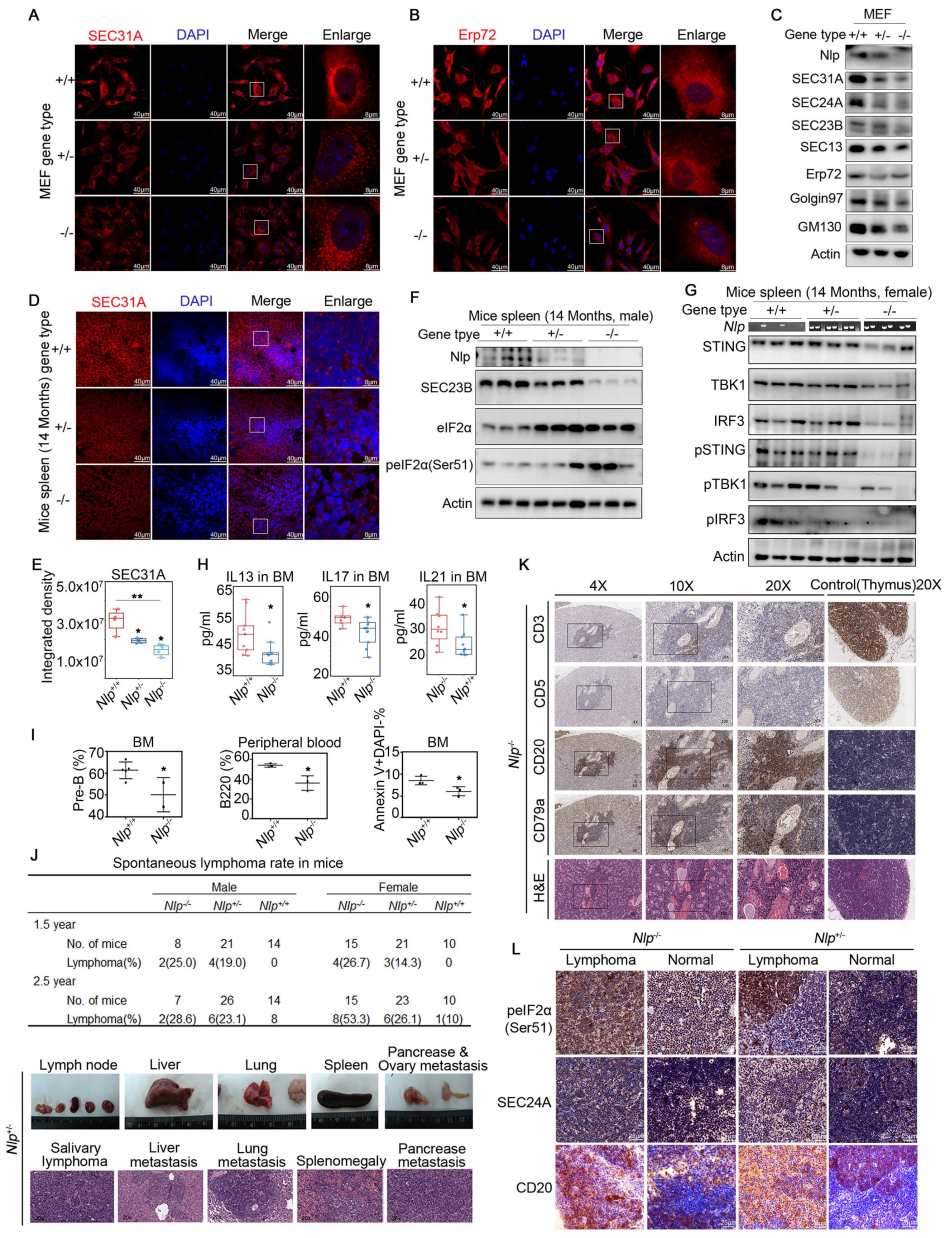
** Nlp caused ER stress and regulated abundance of organelles in Mice model.** (A) Confocal of SEC31A in MEF-*Nlp^+/^*^-^, MEF-*Nlp^-/-^* and MEF-*Nlp^+/+^* cells. (Scale bar, 40 μm or 8 μm.). (B) Confocal of Erp72 in MEF-*Nlp^+/-^*, MEF-*Nlp^-/-^* and MEF-*Nlp^+/+^* cells. (Scale bar, 40 μm or 8 μm.). (C) Immunoblotting of MEF-*Nlp^+/-^*, MEF-*Nlp^-/-^* and MEF-*Nlp^+/+^* cells. (D-E) Confocal of SEC31A in spleens of 14 months *Nlp^+/+^* (n=4), *Nlp^+/-^* (n=4) and *Nlp^-/-^* mice (n=4). (Scale bar, 40 μm or 8 μm. *p<0.05, **p<0.01.). (F) Immunoblotting of 14 months *Nlp^+/+^*, *Nlp^+/-^* and *Nlp^-/-^* male mice spleens. (G) Immunoblotting of 14 months *Nlp^+/+^*, *Nlp^+/-^* and *Nlp^-/-^* female mice spleens. (H) Interleukin levels of *Nlp^-/-^* mice and wildtype mice. IL13 of *Nlp^-/-^* mice (n=10) and wildtype mice (n=10). IL17 of *Nlp^-/-^* mice (n=11) and wildtype mice (n=8). IL21 of *Nlp^-/-^* mice (n=10) and wildtype mice (n=9). (*p<0.05.). (I) Hematologic phenotype of *Nlp^-/-^* mice and wildtype mice. Pre-B rate in bone marrow of *Nlp^-/-^* mice (n=2) and wildtype mice (n=5). B220 rate in peripheral blood of *Nlp^-/-^* mice (n=3) and wildtype mice (n=3). Apoptosis rate in bone marrow of *Nlp^-/-^* mice (n=3) and wildtype mice (n=3). (*p<0.05.). (J) Spontaneous lymphoma incidence of *Nlp^-/-^*, *Nlp^+/-^* and *Nlp^-/-^* mice, and representative images of spontaneous lymphoma in *Nlp^+/-^* mice. (K) H&E and IHC images of spontaneous B cell lymphoma in representative *Nlp^-/-^* mice. (L) IHC images of spontaneous B cell lymphoma and normal lymph node in representative *Nlp^-/-^* and *Nlp^+/-^* mice. (Scale bar, 25 μm.).

Furthermore, when the expression level of Nlp was flattered, distinct changes appeared in morphology between ER and Golgi apparatus using Leica SP8 DIVE and Leica SP8 STED (Fig. 7, I-J). We repeated the ER distribution experiments in MEF-*Nlp*^+/-^, MEF-*Nlp*^-/-^ and the wild type MEF-*Nlp*^+/+^ cells. Consistently, both ER sheets and ER tubes, as well as COPII-coated vesicles and Golgi apparatus decreased with the depletion of Nlp expression (Fig. 8, A-C). Overall, the abnormal expression of Nlp reshaped the communications between organelles and altered protein synthesis and processing. Given that ER-to-Golgi vesicles transport is more plentiful in lymphocytes due to their abundant secretory functions 11, 63, we carried out confocal and immunoblotting assay in the spleen of *Nlp* deficient mice and wide type mice to further explore the levels of vesicle transport in lymphocytes. As expected, deficient Nlp reduced COPII-coated vesicles and decreased the protein expression levels of major COPII vesicle coat components while upregulated UPR to reduce the ER Stress (Fig. 8, D-F). Moreover, we explored the STING-TBK1-IRF3 axis in three gene type mice, and the results suggested that deficient Nlp reduced STING-TBK1-IRF3 axis, which was important innate immune signaling pathway (Fig. 8G). To further validate Nlp could function in vesicles transporting and organelle interacting, we employed livers of the 14-months old *Nlp* knockout mice model and found that COPII-coated vesicles and Golgi exhibited an obvious downregulation in both male and female livers of 14 months *Nlp*^+/-^ and *Nlp*^-/-^ mice compared to *Nlp*^+/+^ control groups (Fig. S6, A-B and D). Then we applied immunoblotting assays in 3 months male *Nlp* deficient mice and wide type mice to exclude aging factor, and there were still markable down regulation of COPII-coated vesicle coat subunits in livers (Fig. S6C). In conclusion, the deficiency of Nlp up-regulated the PERK-eIF2α pathway, and decreased COPII-coated vesicles, STING-TBK1-IRF3 axis as well as Golgi components in cells.

### *Nlp* deficient mice were prone to abnormal vesicle trafficking and spontaneous B cell lymphoma

Given that deficient Nlp reduced COPII-coated vesicles and decreased protein expression levels of major COPII vesicle coat components, while upregulated eIF2α and peIF2α (Ser51) in spleen, we wondered whether the alteration of Nlp influenced the secretion of cytokines in lymphocytes. Interestingly, when we examined the concentrations of main cytokines in bone marrow and peripheral blood in *Nlp* deficiency mice or wildtype mice, we found that statistically reductions in interleukin-13, interleukin-17 and interleukin-21 in bone marrow, while cytokines showed no difference in peripheral blood of *Nlp* deficient mice compared to wildtype mice (Fig. 8H, and Fig. S6, F-G). Interleukin-13, interleukin-17 and interleukin-21 were secreted by immune cells and participated in the development of B cells, especially interleukin-21 could function in B cell selection and differentiation 64-67. We next examined the development of all types of B cells in bone marrow as well as peripheral blood in *Nlp* deficiency mice or wildtype mice (Fig. 8I, and Fig. S6E) and observed that *Nlp* deficiency mice showed reductions of pre-B cells in bone marrow and B cells in peripheral blood, along with a reduction of B cells apoptosis in bone marrow, while there were no differences of other types of B cells in bone marrow. These results suggested that Nlp might regulate the secreted levels of interleukins in bone marrow, which might further influence the development of B cells.

To fully understand the consequence of abnormal B cell development caused by deficiency of Nlp, we made long-term observation in three gene types of mice. We observed that after two periods which end up with 1.5-year and 2.5-year observations in total of 184 mice, *Nlp* deficient mice mainly developed lymphoma (Fig. 8J, and Fig. S6H). In the first period (1.5-year) study, we found 26.1% and 16.7% lymphoma incidence in *Nlp^-/-^* and *Nlp^+/-^* mice respectively, while none of a case of lymphoma in *Nlp^+/+^* mice (p value were 0.0094 and 0.0423). Consistently, in the second period (2.5-year) study, we found 45.5% and 24.5% lymphoma incidence in *Nlp^-/-^* and *Nlp^+/-^* mice respectively, while only 4.2% *Nlp^+/+^* mice were prone to lymphoma (p value were 0.0014 and 0.0486). Immunohistochemical analysis demonstrated *Nlp* deficient mice mainly developed B cell lymphoma, suggesting that abnormal development of B cells caused by *Nlp* deficiency might eventually lead to the development of lymphoma (Fig. 8K, and Fig. S6I). Thus, we further analyzed the levels of SEC24A and peIF2α in lymphoma and lymph node of *Nlp^-/-^* or *Nlp^+/-^* mice respectively, and found that expression levels of both SEC24A and peIF2α were upregulated in lymphoma compared to the normal lymph node, which suggested lymphoma acted more radical in the condition of *Nlp* deficiency (Fig. 8L). Furthermore, single cell analysis also showed deficiency of *Nlp* led to the abnormal secretion of cytokine and partial blockage of B cells development (Fig. S7, A-F).

## Discussion

ER-to-Golgi vesicle trafficking is a continuous and precise process 15, which means there might be a control system to maintain the successive processes of cargoes. However, the constant or changing components during these finely regulated processes remain largely unknown. Therefore, we proposed an adapter-integrated transport model. In this model, particular adapter integrated the whole biological processes of cargoes as an invariable code or marker to maintain the specificity, continuity and accuracy of cargoes. Particular adapters might transport specific cargoes from ER to Golgi independently. Among the adapters, Nlp participated in the forming and transporting of partial ER-to-Golgi vesicles. At the beginning of the vesicle formation, Nlp acted as a platform for the formation of COPII-coated vesicles through interacting directly with SEC31A. At the same time, Nlp could sort or transport cytokinesis and focal adhesion related proteins through binding directly with Rab1B. In addition, Nlp influenced the gathering ability of TFG around COPII-coated vesicles, and affected the transformation of COPII to COPI-coated vesicles. The abnormal expression of Nlp led to alteration of the morphology, distribution and communication manners of ER and Golgi through affecting the ER-to-Golgi trafficking system as well as microtubule networks. Moreover, *Nlp* deficient mice were prone to spontaneous lymphoma, and the abnormal vesicle transport might be an explanation for the development of lymphoma.

To explain ER-to-Golgi intracellular transport, four main models are summarized at present: Vesicular model; Progression model; Diffusion model; Kiss-and-run model 17. In vesicular model, cargoes occur in COPII-coated spherical vesicles, which are transported to Golgi as individual vesicles or vesicle aggregates 68, 69. While in progression model, cargoes in immature ER-to-Golgi carriers are eliminated by retrograde COPI-dependent vesicles 70. In diffusion and kiss-and-run model, cargoes are diffused along constant connections between ER and Golgi or fused by the tubule between ER and Golgi respectively 70, 71. Although these models might have their rationalities under different conditions, there are contradictions for the existence of microtubules during ER-to-Golgi vesicle trafficking, the relationship between COPII and COPI-coated vesicles, or the intracellular distribution of ER, ERES, ERGIC and Golgi. Recently, Weigel et al. pictured the dynamic 3D view of ERESs and found COPII and COPI vesicles acted sequentially in ERESs and delivered cargoes through microtubules to Golgi 15, which solved some contradictions and improved ER-to-Golgi vesicle trafficking model. However, the continuity of cargoes during COPII and COPI-coated vesicles transformation still left unsolved, and therefore our study focused on the integrated transport system that maintained the continuity of multi-step ER-to-Golgi cargo trafficking.

So far, several specific proteins have been found to modulate formation or transport of vesicles between ER and Golgi, including Rabs, SNAREs, cargo receptors, vesicle-coated proteins and multisubunit tethering complexes 17. However, these studies failed to find the maintainer among various regulators involved during ER-to-Golgi multi-step processes. It is worth mentioning that some theories have proposed that adapter could regulate one or some stages during vesicle formation or transport among ER, Golgi, endosome and autophagosome 9, 27, 28, 72-74. Recently, Nlp was found as an adapter that facilitated the movement of dynein-dynactin complexes 33. Nlp is an essential participant of microtubule-associated mission in cells as Nlp plays crucial roles in centrosome maturation, microtubule binding and spindle formation 46, 48. Our latest work demonstrated that Nlp could strengthen the binding ability between Fyco1 and Rab7, two autophagy-related vesicles transporting protein, to facilitate the fusion of autophagosome with lysosome 36. In other work, Nlp also has been proven to function as a platform and linker in both Nlp-Dzank1-Dynein-1 complex and CC2D2A-NINL-MICAL3-RAB8A vesicles in cilia to maintain the normal operation of photoreceptor 37, 46. Therefore, Nlp could interact with different vesicle-binding proteins and sustain the microtubules related transport in different cells. However, the mechanism and capability of Nlp in vesicle networks were not well understood. Interestingly, our current work suggested that Nlp not only functioned as a platform during the formation of COPII-coated vesicles, but also acted as an intermediate medium between vesicle-binding proteins and cell cytoskeleton in ER-to-Golgi vesicle trafficking. Furthermore, our observations in split-TurboID and TurboID assays suggested that specific ERESs cargoes might be selected/transported by different adapters, including Nlp-specific selected/transported proteins such as some cell division or focal adhesion related proteins.

Golgi morphological changes occur significantly in the cases of perturbation of microtubule organization, apoptosis, DNA damage and disease like cancer 75. Previous studies have shown that the abnormal trafficking between ER and Golgi led to ER stress or Golgi morphological changes 58, 76. It's also worth mentioning that overexpression of EF hands domain of Nlp was able to result in fragmented Golgi 77 and microtubule network was changed in the condition of Nlp overexpression 49, 50. Interestingly, the recent research showed that Rab1A reshaped the distribution of ER during mitosis 78. In our current work, we demonstrated that Nlp could interact with Rab1B directly through its EF hands domain, reshape the microtubule networks, and cause Golgi fragmentation. Therefore, our results offered another explanation to the distribution of organelles in cell caused by Nlp overexpression: Nlp might remodel the microtubules through interacting with Rab1B. Meanwhile, deficiency of Nlp would cause ER stress, Golgi fragmentation and also change the communication manner between ER and Golgi. Previous studies have shown that the inhibition of trafficking between ER and Golgi led to ER stress or Golgi morphological changes 58, 79. Thus, the absent of Nlp led to ER-derived vesicle trafficking failure and caused Golgi fragmentation in a way of passive adaptation. Particularly, when Nlp was downregulated, PERK-eIF2α pathway was aroused without the activation of CHOP to survive the cells as a protective factor. Thus, Nlp appeared to maintain cell viability by altering the way ER and Golgi communicated.

It has been reported that alterations of vesicle trafficking and ER stress were closely related to the development of tumors 80-84. The abruption of ER-to-Golgi vesicle trafficking plays essential roles in lymphoma as the secretory proteins delivered by ER-to-Golgi vesicles functioned in lymphocyte development 84, 85. Interestingly, our findings demonstrated the critical roles of Nlp in B cell development through affecting levels of interleukin-13,17 and 21 secreted by immune cells, and *Nlp* deficiency mice were prone to B cell lymphoma. Moreover, PERK-eIF2α pathway was further upregulated in lymphoma of *Nlp^-/-^* and* Nlp^+/-^* mice compared to normal lymph node. Here we think higher level of ER stress will lead to the vulnerability of tumor, which offers a possibility for the development of novel cancer treatment.

In summary, we demonstrated that Nlp, as a particular adapter in the process of ER-to-Golgi vesicle transport, functioned in the assembly, budding, and transporting of partial ER-to-Golgi vesicles that might contain cytokinesis and focal adhesion related proteins. Perturbation of Nlp led to ER stress and Golgi fragmentation, changing the communication mode of ER and Golgi. Therefore, Nlp was crucial in maintaining cellular homeostasis through sustaining the correct maturation of proteins, proper distributions of organelles as well as proteins, moderate cell stiffness and accurate signal transductions.

## Supplementary Material

Supplementary figures and tables, movie legends.

Supplementary videos.
