# Supplementary material for: Nlp-dependent ER-to-Golgi transport
Source: Int J Biol Sci. 2024 May 11;20(8):2881–903. doi: 10.7150/ijbs.91792 (PMC11186355; doi:10.7150/ijbs.91792)
Supplement: Supplementary file 1 — Supplementary figures and tables, movie legends. [file ijbsv20p2881s1.pdf]

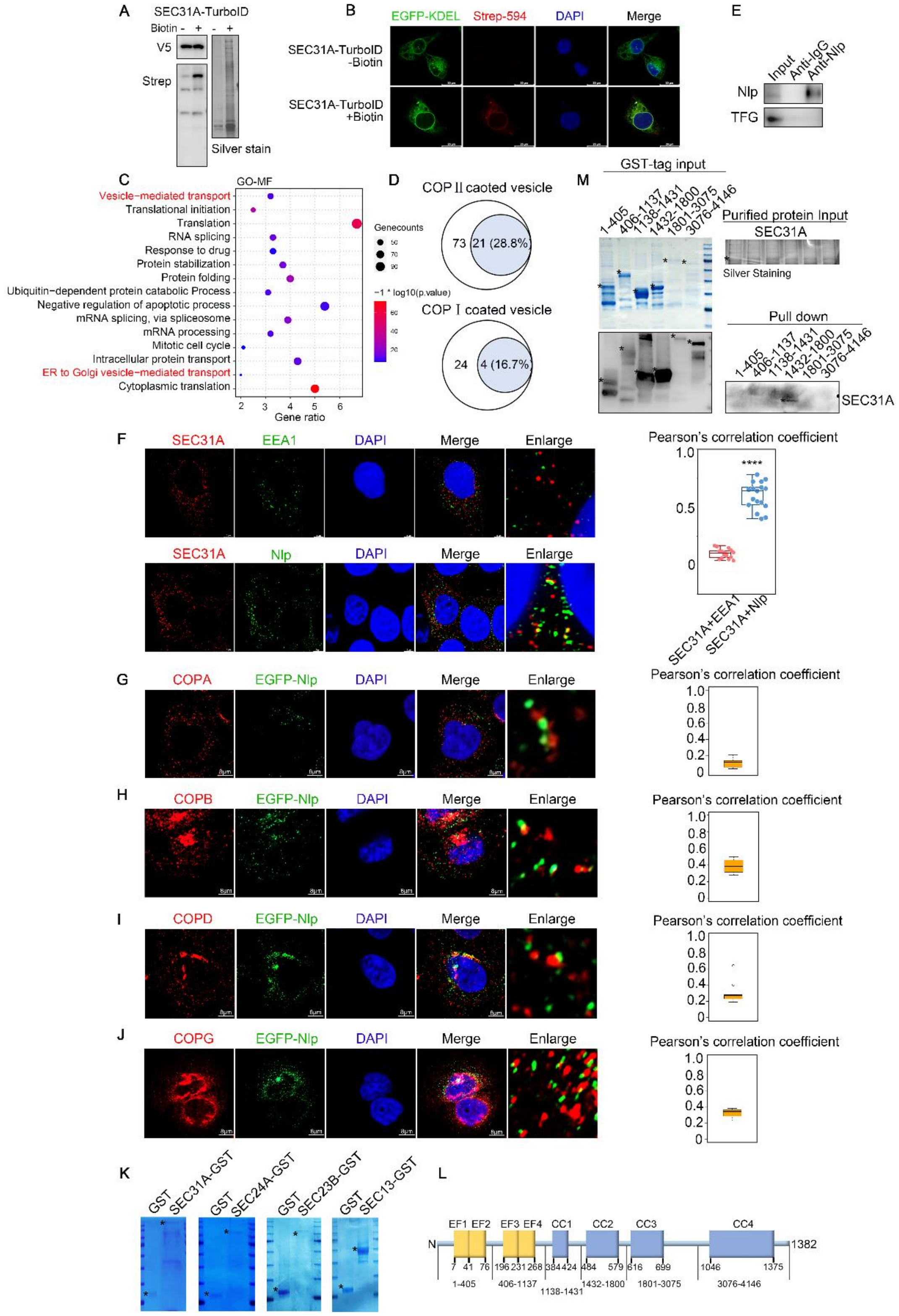


**Figure S1. Nlp interacted with ER-to-Golgi vesicles and altered their stability.**

(A) Immunoblotting and silver staining assay in HeLa cells that have been transfected with SEC31A-TurboID-V5 plasmids for 36 hours. Cells were labeled with or without 50μM biotin for 10 min before washing and collecting.

(B) Confocal of SEC31A-TurboID-V5 system. HeLa-EGFP-KDEL Cells transfected with SEC31A-TurboID-V5 plasmids were treated with or without 50 μM biotin for 10 min before fixing. (Scale bar, 20 μm.).

(C-D) Enrichment analysis of Molecular Functions in Nlp-interacted proteins using two different Nlp antibodies. Ranked by p-value and cut off by minimal percentage of 2%.

(E) Co-IP assay using anti-Nlp or anti-IgG in HeLa cells.

(F) Colocalization of SEC31A and EEA1 or EGFP-Nlp by Leica. Pearson's correlation coefficient was analyzed. (Scale bar, 5 μm. ****p<0.0001.).

(G-J) Colocalization of Nlp and (G) COPA, (H) COPB, (I) COPD or (J) COPG by LSM980. Pearson's correlation coefficient was analyzed. (Scale bar, 8 μm.).

(K) Coomassie stain gel of GST, SEC31A-GST, SEC24A-GST SEC23B-GST or SEC13-GST purified protein related to Figure 2 A-D.

(L) Indicated truncates of Nlp were constructed according to its functional domains.

(M) SEC31A purified protein was obtained from SEC31A-GST using Thrombin. After verifications by Coomassie brilliant blue, silver staining and Western blot, GST pull-down assay was performed using GST-tag purified proteins of Nlp truncates and SEC31A purified protein.

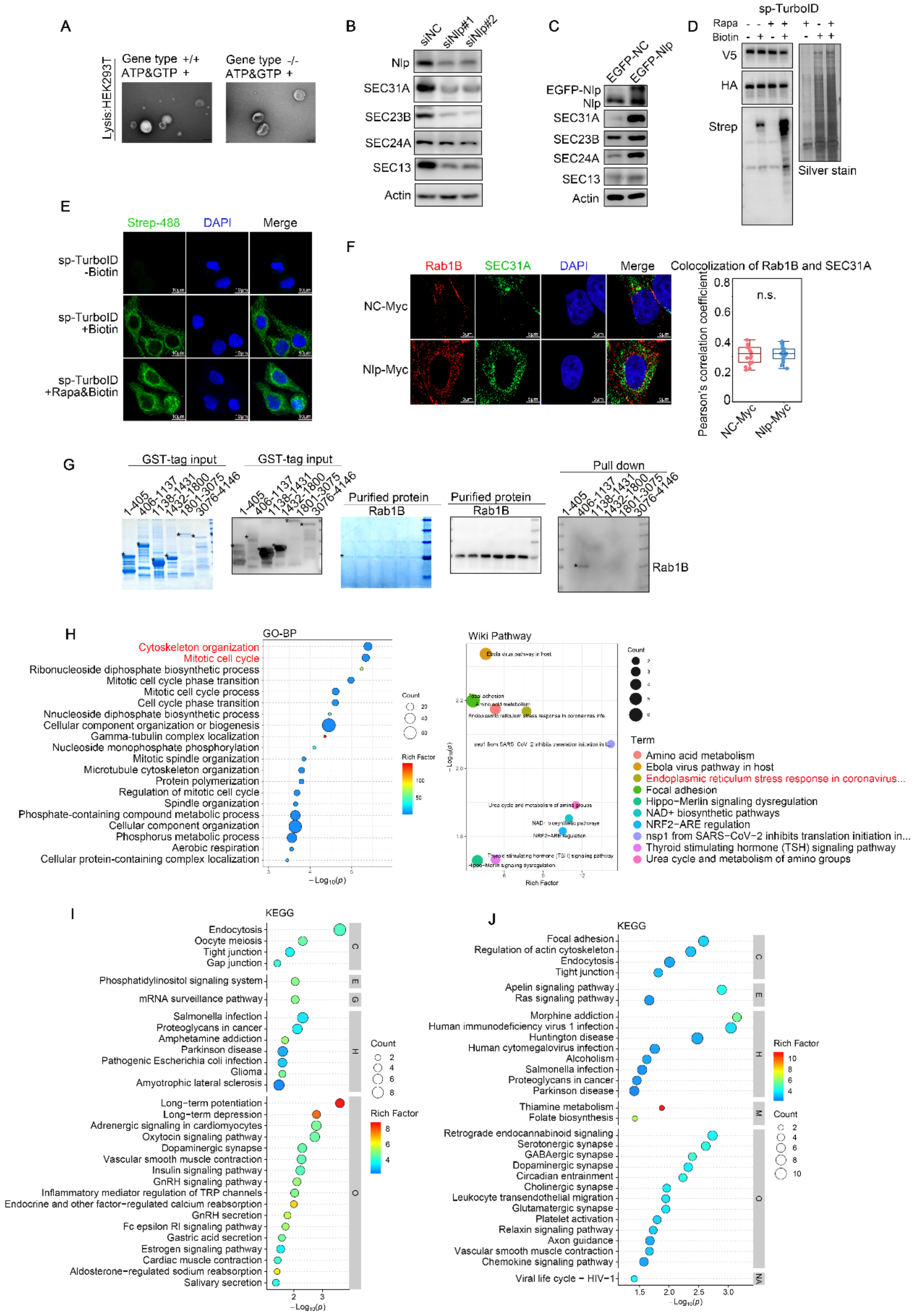


**Figure S2. TurboID and Split-TurboID system involved to analyze Nlp-associated transporting cargoes.**

(A) Negative staining TEM visualization of the morphology of the vesicle membrane structures formed by *in vitro* vesicle formation assay. Membrane source was from MEF-*Nlp*$^{-/-}$ or MEF-*Nlp*$^{+/+}$ cells, and cytosolic protein source was HEK293T cell lysis. (Scale bar,100 nm.).

(B) Immunoblotting of HeLa cells that have been transfected with siRNA for 48 hours.

(C) Immunoblotting of stable EGFP-NC HeLa cells and EGFP-Nlp HeLa cells.

(D) Immunoblotting and silver staining assay in HeLa cells that have been transfected with Split-TurboID system plasmids for 36 hours. Cells were labeled without Biotin, with 50 μM biotin or with 50 μM biotin & 100 nM rapamycin for 4 hours before washing and collecting.

(E) Confocal of Split-TurboID system. HeLa cells transfected with Split-TurboID system for 36 hours were treated with or without 50 μM biotin for 1 hour before fixing. (Scale bar, 10 μm.).

(F) Colocalization of SEC31A and Rab1B by LSM980 in HeLa cells that have been transfected with plasmids for 48 hours. (Scale bar, 5 μm. n.s. represented no significance.).

(G) Rab1B purified protein was obtained from Rab1B-GST using Thrombin. After verifications by Coomassie brilliant blue and Western blot, GST pull-down assay was performed using GST-tag purified proteins of Nlp truncates and Rab1B purified protein.

(H) Results of SEC31A-TurboID and Split-TurboID were grouped into three parts: the intersection of the TurboID and split-TurboID as Nlp-specific sorting/transporting proteins; the remaining part of TurboID as non-Nlp-specific sorting/transporting proteins; the remaining part of split-TurboID as Nlp-associated proteins. Figures represented enrichment analysis of Biology Process and WiKi Pathway of Nlp-associated proteins.

(I-J) Results of SEC31A-TurboID and Split-TurboID system. Figures represented enrichment analysis of KEGG Pathway of (I) non-Nlp specific sorting/transporting proteins, (J) Nlp-specific sorting/transporting proteins.

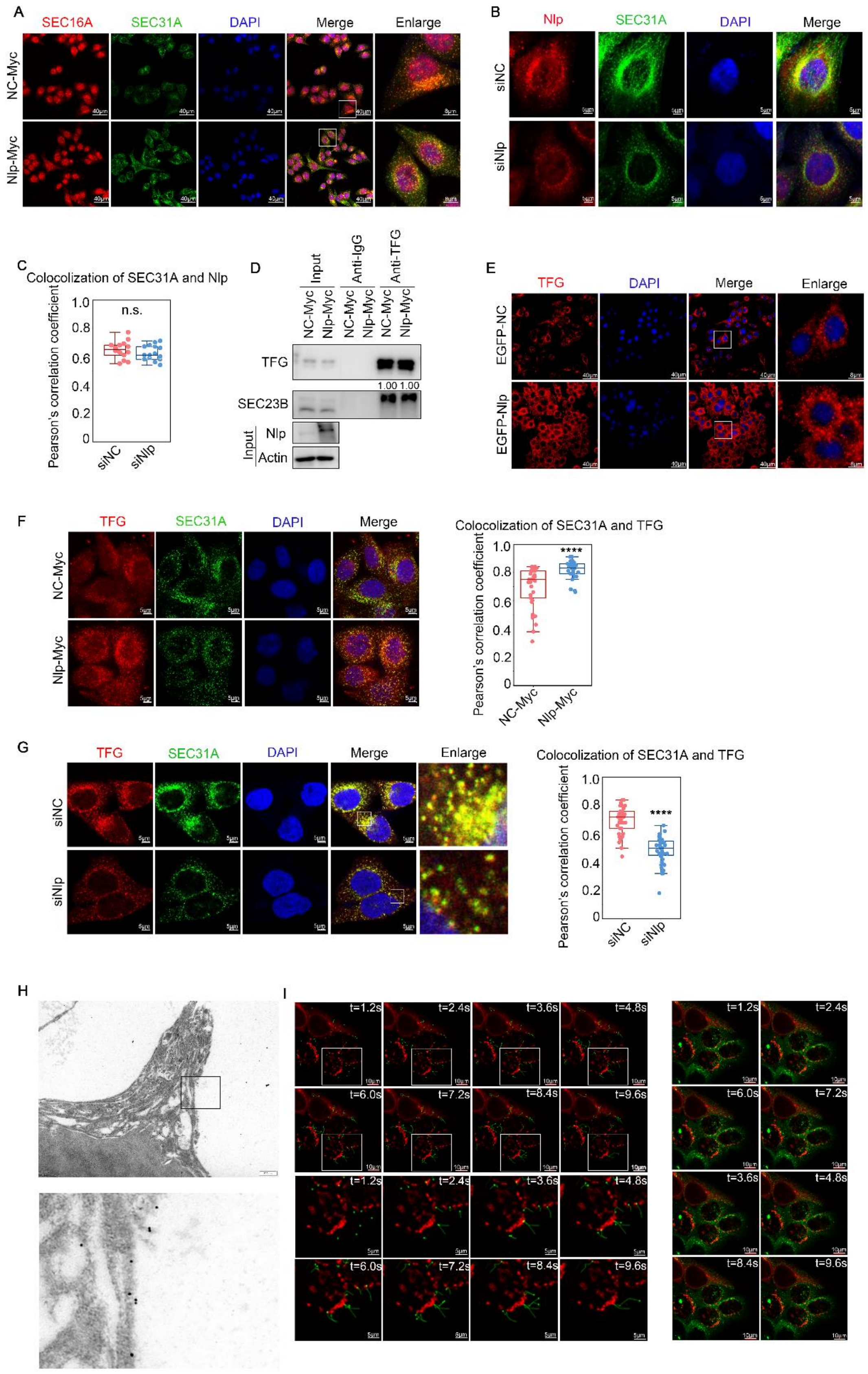


**Figure S3. Nlp regulated budding and uncoating of COPII-coated vesicles.**

(A) Colocalization of Nlp and SEC16A by LSM780 in HeLa cells that have been transfected with siRNA for 48 hours. (Scale bar, 40 μm or 8 μm.).

(B-C) Colocalization of Nlp and SEC31A by LSM780 in HeLa cells that have been transfected with siRNA for 48 hours. (Scale bar, 5 μm. n.s. represented no significance.).

(D) Co-IP assay using anti-TFG or anti-IgG in HeLa cells that have been transfected with plasmids for 48 hours.

(E) Confocal assay represented the aggregation ability of TFG in HeLa cells that have been transfected with plasmids for 48 hours. (Scale bar, 40 μm or 8 μm.).

(F-G) Colocalization of TFG and SEC31A by LSM780 in HeLa cells that have been transfected with plasmids or siRNA for 48 hours. (Scale bar, 5 μm. ****$p<0.0001$.).

(H) Immunogold TEM labeled by anti-Nlp in HeLa cells. (Scale bar, 500 nm.).

(I) Time lapse of EGFP-Nlp and Golgi-mCherry. (Scale bar, 10 μm or 5 μm.).

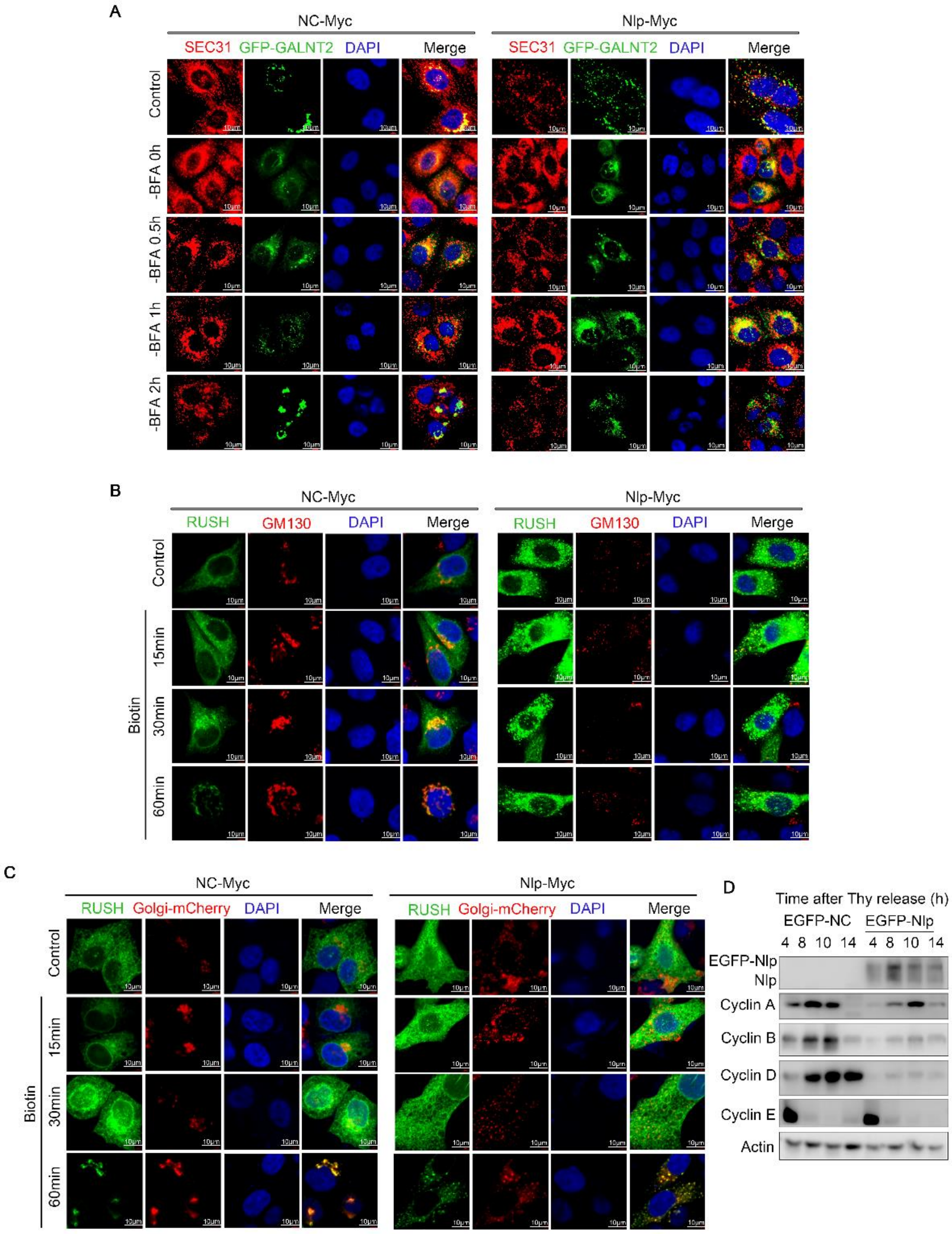


**Figure S4. Overexpression of Nlp induced fragmentation of Golgi and changed ER-Golgi vesicle trafficking manner.**

(A) Colocalization of GFP-GALNT2 and SEC31A by LSM780 in HeLa cells that have been transfected with GFP-GALNT2 and Nlp-Myc or NC-Myc for 36 hours. After the transfection, cells were treated with 5 µg/mL BFA for 30 min, and removed BFA for different times to capture the movements of vesicles. (Scale bar, 10 µm.).

(B-C) Confocal for RUSH assay. (B) HeLa cells or (C) stable Golgi-mCherry HeLa cells were transfected with Str-KDEL-SBP-GFP plasmid for 36 hours, and then incubated with 80 μM of biotin for different time (0~60 min) for confocal assay. (Scale bar, 10 μm.).

(D) Stable control HeLa-C3 or HeLa-EGFP-Nlp cells were arrested in G1-S boundary and released into fresh medium. Cells were collected every 4 hours for confocal (Figure 6F), and verified by immunoblotting.

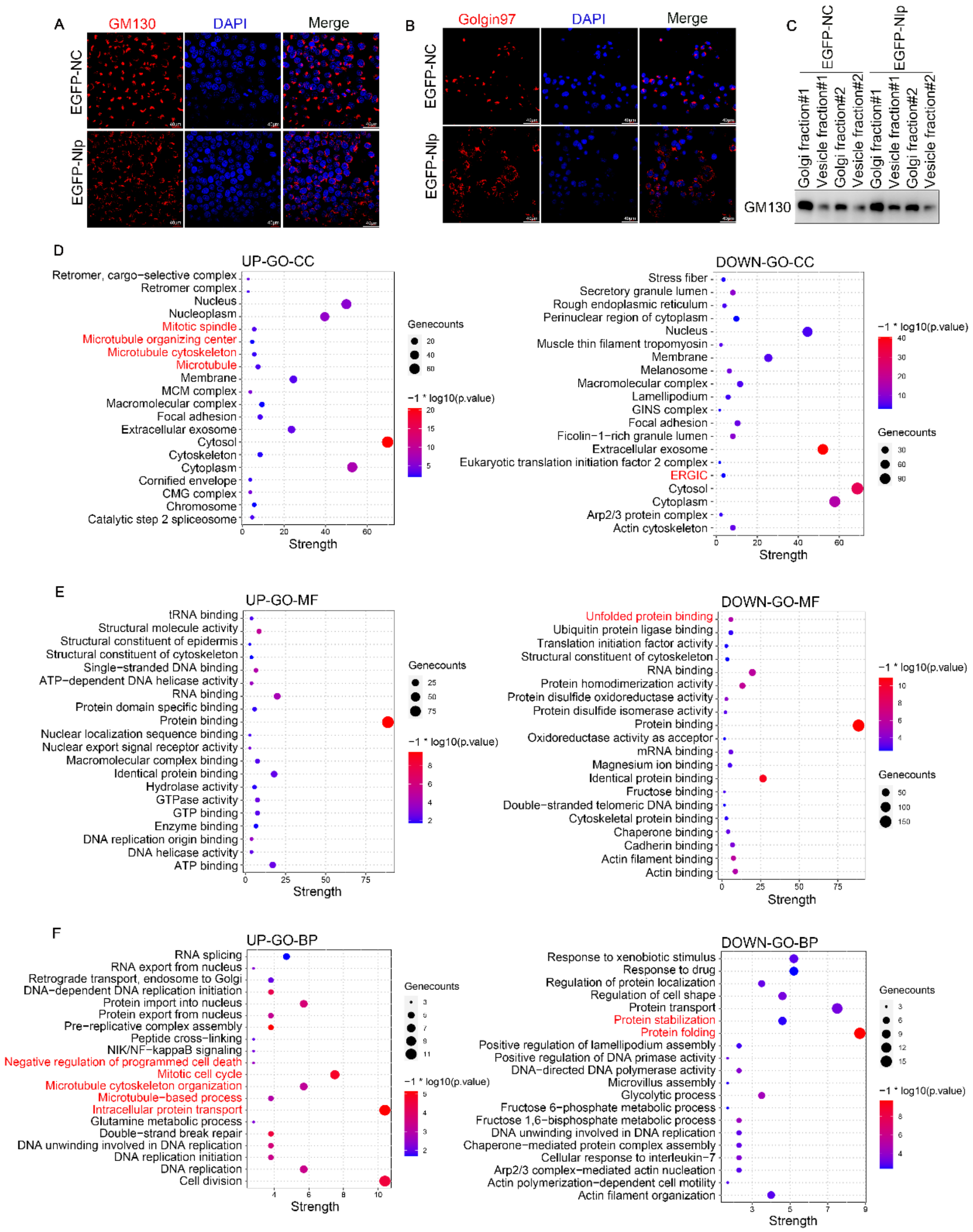


**Figure S5. Overexpression of Nlp induced functional fragmentation of Golgi reduced ERGIC component as well as protein stabilization.**

(A-B) Confocal of (A) GM130 and (B) Golgin97 in stable EGFP-NC HeLa cells and EGFP-Nlp HeLa cells. (Scale bar, 40 µm.).

(C) Golgi fractions and secretory vesicle fractions were purified from stable control HeLa-C3 or HeLa-EGFP-Nlp cells, and verified by immunoblotting.

(D-F) Golgi apparatus was purified from stable control HeLa-C3 or HeLa-EGFP-Nlp cells for LC-MS/MS. Figures represented the enrichment analysis of upregulated proteins and downregulated protein when Nlp was overexpressed compared to control group (FC=2).

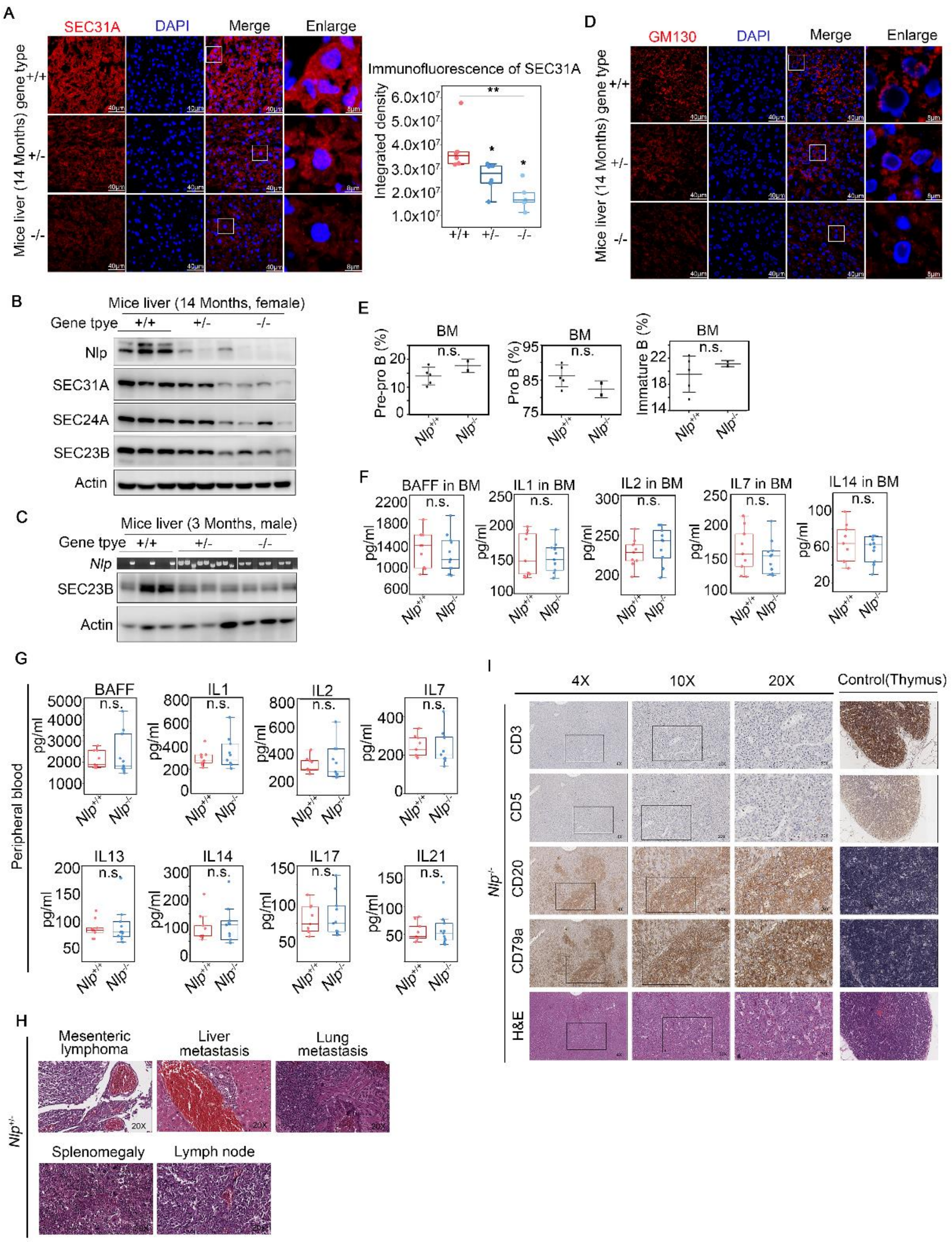

**Figure S6.** ***Nlp*** **deficient mice displayed spontaneous lymphoma.**

(A) Confocal of SEC31A in livers of 14 months $Nlp^{+/+}$ (n=6), $Nlp^{+/-}$ (n=6) and $Nlp^{-/-}$ mice (n=5). (Scale bar, 40 μm or 8 μm. *p<0.05, **p<0.01.).

(B) Immunoblotting of 14 months $Nlp^{+/+}$, $Nlp^{+/-}$ and $Nlp^{-/-}$ female mice livers.

(C) Immunoblotting of 3 months $Nlp^{+/+}$, $Nlp^{+/-}$ and $Nlp^{-/-}$ male mice livers.

(D) Confocal of GM130 in livers of 14 months *Nlp*$^{+/+}$, *Nlp*$^{+/-}$ and *Nlp*$^{-/-}$ mice. (Scale bar, 40 μm or 8 μm.).

(E) Hematologic phenotype of *Nlp*$^{-/-}$ (n=2) mice and wildtype mice (n=5). (n.s. represented no significance.).

(F-G) Interleukin levels of *Nlp*$^{-/-}$ mice (n=11) and wildtype mice (n=10). (n.s. represented no significance.).

(H) Representative images of spontaneous lymphoma in *Nlp*$^{+/-}$ mice.

(I) H&E and IHC images of spontaneous lymphoma in representative *Nlp*$^{-/-}$ mice. Control group was same with Figure 8K.

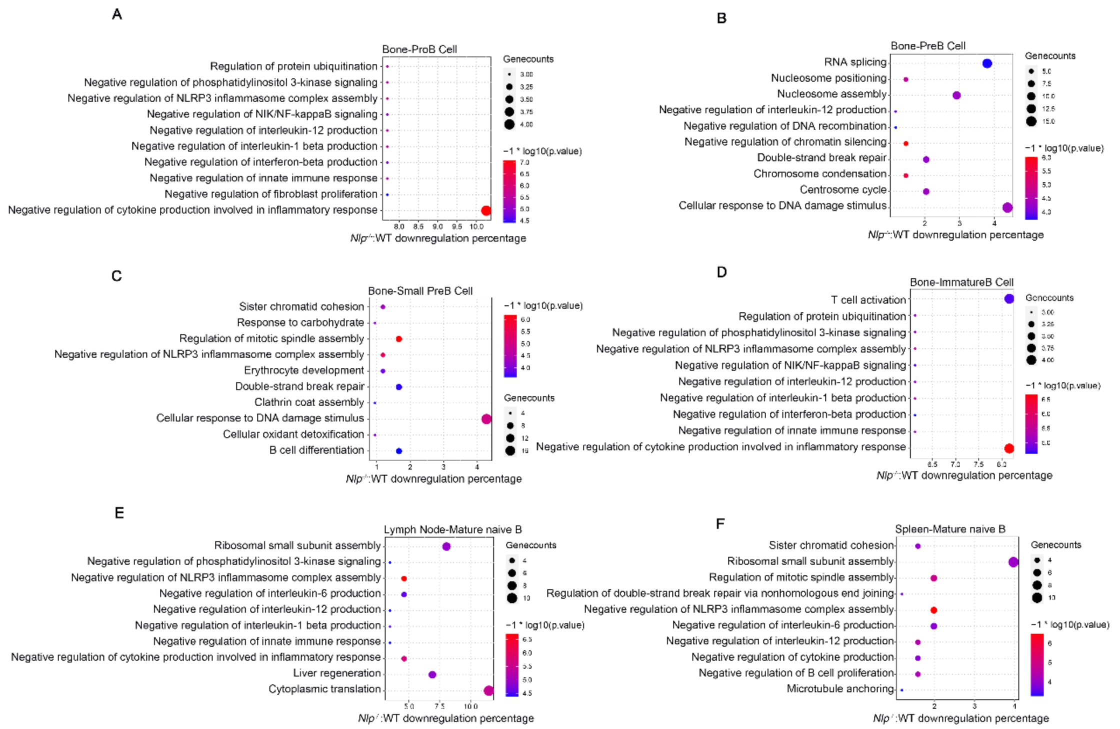


**Figure S7. Single cell analysis of *Nlp* deficient mice and wildtype mice.**

(A-D) GO analysis of downregulation of (A) Pro-B cells, (B) Pre-B cells, (C) small Pre-B cells and (D) immature B cells in bone marrow of *Nlp*$^{-/-}$ mice compared to wildtype mice.

(E) GO analysis of downregulation of mature naive B cells in lymph node of *Nlp*$^{-/-}$ mice compared to wildtype mice.

(F) GO analysis of downregulation of mature naive B cells in spleen of *Nlp*$^{-/-}$ mice compared to wildtype mice.

**Movie S1. Two-color three-dimensional N-STORM images of Nlp (red) and SEC31A (green) localizations in the HeLa cells.**

**Movie S2. Two-color three-dimensional N-STORM images of Nlp (red) and SEC24A (green) localizations in the HeLa cells.**

**Movie S3. FRAP of EGFP-TFG in HeLa cells.** Cells that have been transfected with NC-Myc or Nlp-Myc plasmids for 36 hours. The intensity of EGFP-TFG was decreased to 20% using photobleaching and observed its recovery ability for 200s.

**Movie S4. Time lapse movement recording of RUSH in HeLa cells.** Cells that have been transfected with Str-KDEL-SBP-GFP and NC-Myc or Nlp-Myc plasmids for 36 hours, and then incubated with 80 μM of biotin to detect movement.

**Movie S5. Time lapse movement recording of RUSH in HeLa cells.** Cells that have been transfected with Str-KDEL-SBP-GFP plasmids and siNC or siNlp for 36 hours, and then incubated with 80 μM of biotin to detect movement.

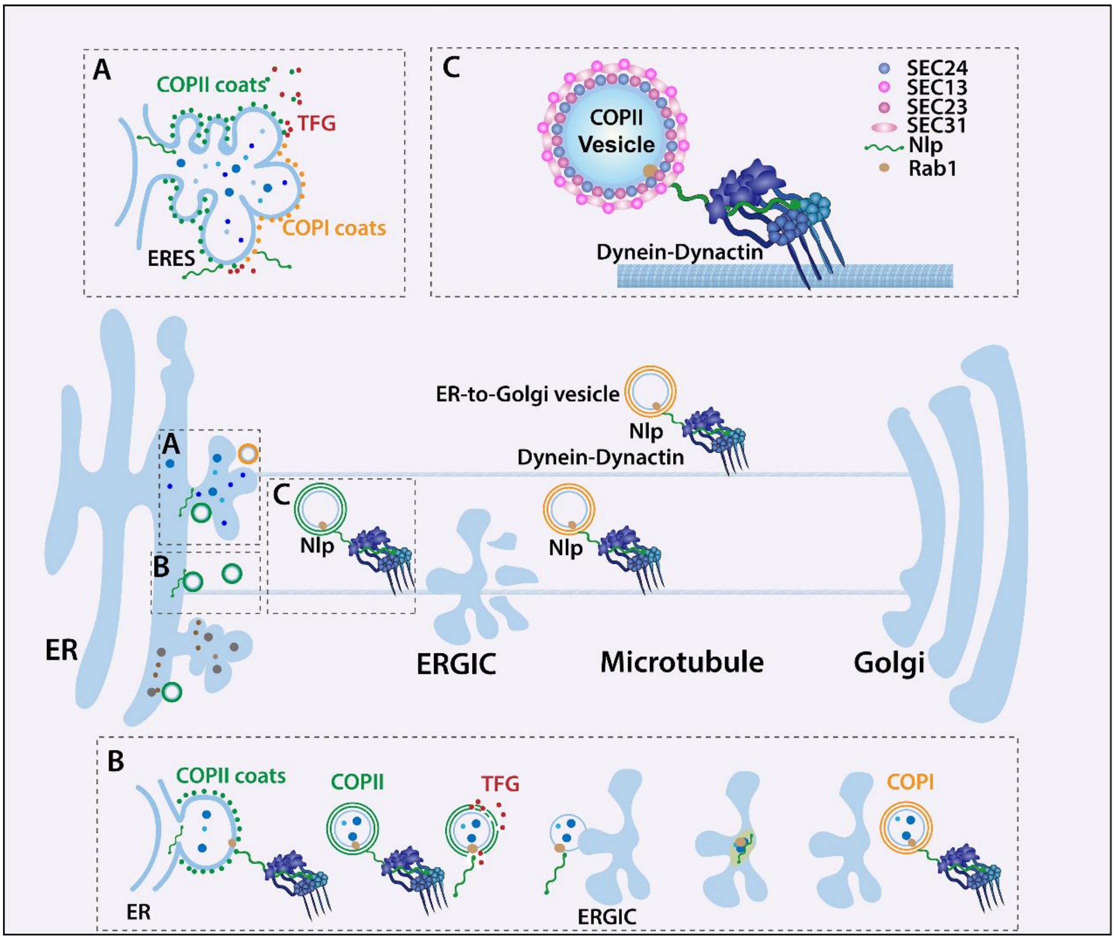


**Graphical abstract. Adapter integrated ER-to-Golgi vesicle transport model.** Particular adapter integrated the whole biological processes of cargoes as an invariable code or marker to maintain the specificity, continuity and accuracy of cargoes. Among the adapters, Nlp participated in the forming and transporting of partial ER-to-Golgi vesicles. At the beginning of the vesicle formation, Nlp acted as a platform for the assembly of COPII-coated vesicles, and might participate in cargo selection. During transporting, Nlp cooperated with Rab1 to regulate the continuity of COPII and COPI-coated vesicles transformation, the direction of partial ER-to-Golgi vesicles, as well as the stability of vesicles and transport system.

**Supplementary Table 1. Key resources table.**

| REAGENT or RESOURCE | SOURCE | IDENTIFIER |
|---|---|---|
| Antibodies | | |

| | | |
|---|---|---|
| Sec31A rabbit polyclonal antibody | Proteintech | Cat#17913-1-AP |
| Sec31A mouse monoclinal antibody | Santa Cruz | Cat#sc-376587 |
| Sec24A rabbit polyclonal antibody | Proteintech | Cat#15958-1-AP |
| Sec24A mouse monoclinal antibody | Santa Cruz | Cat#sc-517155 |
| Sec13 rabbit polyclonal antibody | Proteintech | Cat#15397-1-AP |
| Sec13 mouse monoclinal antibody | Santa Cruz | Cat#sc-514308 |
| Sec23B rabbit polyclonal antibody | Abcam | Cat#ab151258 |
| TFG mouse monoclinal antibody | proteintech | Cat#66916-1-Ig |
| TFG rabbit polyclonal antibody | Proteintech | Cat#11571-1-AP |
| GALNT rabbit polyclonal antibody | Sigma-Aldrich | Cat#HPA011222 |
| GM130 rabbit polyclonal antibody | Abcam | Cat#ab52649 |
| GM130 mouse monoclinal antibody | Abcam | Cat#ab169276 |
| Golgin97 mouse monoclinal antibody | Abcam | Cat#ab169287 |
| Erp72 rabbit polyclonal antibody | Proteintech | Cat#14712-1-AP |
| Erp72 mouse monoclinal antibody | BD | Cat#610970 |
| Sec61B rabbit polyclonal antibody | CST | Cat#14648S |
| Sec16A rabbit polyclonal antibody | Proteintech | Cat#20025-1-AP |
| NINL rabbit polyclonal antibody | Sigma-Aldrich | Cat#HPA000686 |
| Nlp rabbit polyclonal antibody | MBL | N/A |
| COPG rabbit polyclonal antibody | Proteintech | Cat#12393-1-AP |
| beta COP rabbit polyclonal antibody | Abcam | Cat#ab2899 |
| COPA mouse monoclinal antibody | Santa Cruz | Cat#sc-398099 |
| COPB mouse monoclinal antibody | Santa Cruz | Cat#sc-393615 |

| | | |
|---|---|---|
| COPD mouse monoclinal antibody | Santa Cruz | Cat#sc-515549 |
| STX5 mouse monoclinal antibody | Santa Cruz | Cat#sc-365124 |
| Rab1B rabbit polyclonal antibody | Proteintech | Cat#17824-1-AP |
| DYNC1H1 Polyclonal Antibody | Proteintech | Cat#12345-1-AP |
| P150 Glued Polyclonal Antibody | Proteintech | Cat#55182-1-AP |
| PERK rabbit polyclonal antibody | Proteintech | Cat#20582-1-AP |
| eIF2α rabbit polyclonal antibody | CST | Cat#5324S |
| CHOP rabbit polyclonal antibody | Proteintech | Cat#15204-1-AP |
| peIF2α rabbit polyclonal antibody | CST | Cat#3398S |
| ATF6 rabbit polyclonal antibody | Proteintech | Cat#24169-1-AP |
| FAM134B rabbit polyclonal antibody | Proteintech | Cat#21537-1-AP |
| IRE1α rabbit polyclonal antibody | Proteintech | Cat#27528-1-AP |
| Sec62 rabbit polyclonal antibody | abcam | Cat#ab244335 |
| CD3e rabbit monoclonal antibody | ThermoFisher | Cat#MA5-14524 |
| CD5 rat monoclonal antibody | ThermoFisher | Cat#MA5-17781 |
| CD20 rabbit monoclonal antibody | ThermoFisher | Cat#PA5-16701 |
| CD79a rabbit monoclonal antibody | ThermoFisher | Cat#MA5-14556 |
| α-Tubulin mouse monoclonal antibody | Proteintech | Cat#66031-1-Ig |
| β-Actin mouse monoclonal antibody | CST. | Cat#3700 |
| HA-Tag rabbit polyclonal antibody | CST | Cat#3724S |
| V5-Tag rabbit polyclonal antibody | CST | Cat#13202S |
| His mouse monoclonal antibody | Santa Cruz | Cat#sc-8036 |
| GST mouse monoclonal antibody | Santa Cruz | Cat#sc-138 |

| | | |
|---|---|---|
| TMEM173/STING polyclonal antibody | Proteintech | Cat#19851-1-AP |
| Phospho-STING (Ser366) antibody | CST | Cat#85735 |
| TBK1/NAK (D1B4) antibody | CST | Cat#3504 |
| Phospho-TBK1/NAK (Ser172) antibody | CST | Cat#5483 |
| IRF-3 (D83B9) antibody | CST | Cat#4302 |
| Phospho-IRF-3 (Ser396) antibody | CST | Cat#4947 |
| SREBP1 rabbit polyclonal antibody | abcam | Cat#ab28481 |
| VEGFA rabbit polyclonal antibody | abcam | Cat#ab52917 |
| beta Catenin polyclonal antibody | Invitrogen | Cat#71-2700 |
| Streptavidin, Alexa Fluor 488 conjugate | ThermoFisher | Cat#S11223 |
| Goat-anti-Rabbit, Alexa Fluor 647 | Beyotime Bio. | Cat#A0468 |
| Goat-anti-Mouse, Alexa Fluor 647 | Beyotime Bio. | Cat#A0473 |
| Goat-anti-Rabbit, Alexa Fluor 488 | Beyotime Bio. | Cat#A0428 |
| Goat-anti-Mouse, Alexa Fluor 488 | Beyotime Bio. | Cat#A0423 |
| Goat-anti-Rabbit, Alexa Fluor 594 | ZSGB-BIO | Cat#ZF-0516 |
| Goat-anti-Rabbit IgG HRP-polymer | ZSGB-BIO | Cat#PV-6001 |
| Goat-anti-Mouse IgG HRP-polymer | ZSGB-BIO | Cat#PV-6002 |
| Bacterial and virus strains | | |
| BL21(DE3) Chemically Competent Cell | TransGen Biotech | Cat#CD601-02 |
| Trans5α Chemically Competent Cell | TransGen Biotech | Cat#CD201-01 |
| Chemicals, peptides, and recombinant proteins | | |
| Fetal Bovine Serum | Gemini | Cat#A87F82H |
| OptiPrep™ Density Gradient Medium | Sigma-Aldrich | Cat#D1556 |

| | | |
|---|---|---|
| ATP (100mM, Nuclease free) | Beyotime Bio. | Cat#D7378 |
| GTP (100mM, Nuclease free) | Beyotime Bio. | Cat#D7380 |
| Creatine phosphate | Solarbio | Cat#C9921 |
| Creatine phosphokinase | Solarbio | Cat#C9470 |
| HEPES | ThermoFisher | Cat#15630106 |
| PIPES | Beyotime Bio. | Cat#ST470 |
| EGTA | Beyotime Bio. | Cat#ST068 |
| 0.25M $CaCl_2$ | Beyotime Bio. | Cat#ST365 |
| Digitonin | Sigma-Aldrich | Cat#D141 |
| Paclitaxel | Selleck | Cat#S1150 |
| Polybrene | Genechem | Cat#REVG0001 |
| Lipofectamine 2000 | ThermoFisher | Cat#11668019 |
| Neofect | Neofect | Cat#TF201201 |
| Biotin | Sigma-Aldrich | Cat#B4501 |
| Tunicamycin | MCE | Cat#HY-A0098 |
| GSK2606414 | Selleck | Cat#S7307 |
| Nocodazole | MCE | Cat#HY-13520 |
| TPE-MI | MCE | Cat# HY-143218 |
| Trimethoprim | MCE | Cat#HY-B0510 |
| Brefeldin A | Selleck | Cat#S7046 |
| Thermo Scientific Pierce Beads | ThermoFisher | Cat#88816 |
| Glutathione High Capacity Magnetic Agarose Beads | Sigma-Aldrich | Cat#G0924 |
| Protein A/G Magnetic Beads | MCE | Cat#HY-K0202 |

| | | |
|---|---|---|
| Experimental models: Cell lines | | |
| HeLa cells | This paper | N/A |
| KYSE450 cells | This paper | N/A |
| *Nlp*$^{+/+}$ MEF cells | This paper | N/A |
| *Nlp*$^{+/-}$ MEF cells | This paper | N/A |
| *Nlp*$^{-/-}$ MEF cells | This paper | N/A |
| Experimental models: Organisms/strains | | |
| *Nlp*$^{+/+}$ C57BL/6 | In house | N/A |
| *Nlp*$^{+/-}$ C57BL/6 | In house | N/A |
| *Nlp*$^{-/-}$ C57BL/6 | In house | N/A |
| Oligonucleotides | | |
| siRNA, see Table S2 | This paper | N/A |
| qPCR primer, see Table S2 | This paper | N/A |
| Recombinant DNA | | |
| Str-KDEL_ST-SBP-EGFP | (Franck Perez., 2012) | Addgene#65264 |
| C1(1-29)-TurboID-V5_Pcdna3 | (Alice Ting., 2018) | Addgene#107173 |
| pLX304 CMV FKBP-V5-sTurboID (N) | (Alice Ting., 2020) | Addgene#153002 |
| pLX304 CMV HA-HaloTag-FRB-sTurboID | (Alice Ting., 2020) | Addgene#153003 |
| TOP-H2B-YFP-DD | (Han et al., 2014) | Addgene#96891 |
| SEC31A-TurboID-V5_Pcdna3 | This paper | N/A |
| pLX304 CMV HA-HaloTag-Sec31A-FRB-split-TurboID(C) | This paper | N/A |

| | | |
|---|---|---|
| pLX304 CMV NINL-FKBP-split-TurboID(N) | This paper | N/A |
| pEGFP-C3-Nlp | This paper | N/A |
| pEGFP-C3-NC | This paper | N/A |
| Golgi-mCherry | Inovogen | Cat#KLV3500 |
| pCS+MT-Nlp-Myc | This paper | N/A |
| pCS+MT-NC-Myc | This paper | N/A |
| pEGFP-TFG | This paper | N/A |
| GFP-GalNAc-T2 | SinoBio | Cat#HG13764 |
| pET28A-Nlp-6*His | This paper | N/A |
| pGEX-4T-1-SEC31A | This paper | N/A |
| pGEX-4T-1-SEC24A | This paper | N/A |
| pGEX-4T-1-SEC23B | This paper | N/A |
| pGEX-4T-1-SEC13 | This paper | N/A |
| pGEX-4T-1-Rab1B | This paper | N/A |
| Software and algorithms | | |
| GraphPad Prism | GraphPad Software | |
| R | R project | |
| ImageJ (Fiji) | NIH | |
| FlowJo | FlowJo | |
| Nikon Elements | Nikon | |
| ZEN Zeiss | Zeiss | |
| OriginPro | OriginLab | |

**Supplementary Table 2. List of oligonucleotides used in this study.**

| Name | Sequence (5’-3’) |
|---|---|
| si-h-NINL_101 | CGCGCTACTTCTAGAGTCT |
| si-h-NINL_102 | GGAGTTCCATAGACTTTCT |
| si-h-NINL_103 | AGTTCGCGCTCTCAACAAA |
| si-h-SEC31A_101 | GATGCAACATTTAGTACGA |
| si-h-SEC31A_102 | GCATGGCCGATGCCATTAT |
| si-h-SEC31A_103 | GGACACTAGTACTGTAGGA |
| m-NINL-1-F | CTCCCCTCCCCATTTTCTTAC |
| m-NINL-2-R | CAGTGGTCCAGGCTCTAGTTTTG |
| m-NINL-3-F | GAGGGTTTATTGGATACACGG |
| m-NINL-4-R | AACAGCACCTTTGATGCCTAC |
| m-NINL-1a-F | TGGGATCTGATTCCCTCTTCT |
